# Urban and nomadic isotopic niches reveal dietary connectivities along Central Asia’s Silk Roads

**DOI:** 10.1038/s41598-018-22995-2

**Published:** 2018-03-26

**Authors:** Taylor R. Hermes, Michael D. Frachetti, Elissa A. Bullion, Farhod Maksudov, Samariddin Mustafokulov, Cheryl A. Makarewicz

**Affiliations:** 1 Institute for Prehistoric and Protohistoric Archaeology, Christian-Albrechts-Universität zu Kiel, Johanna-Mestorf-Straße 2-6, 24118 Kiel, Germany; 2 Graduate School ‘Human Development in Landscapes’, Christian-Albrechts-Universität zu Kiel, Leibniz Straße 3, 24118 Kiel, Germany; 3 SAIE Laboratory, Department of Anthropology, Washington University in St. Louis, CB 1114, One Brookings Drive, St. Louis, MO 63130 USA; 4 Institute for Archaeological Research, Academy of Sciences of Uzbekistan, Yahya Gulamov Street No. 70, Tashkent, 100000 Uzbekistan; 5 Afrasiyab Museum, Islam Karimov Street No. 7, Samarkand, 114151 Uzbekistan

## Abstract

The ancient ‘Silk Roads’ formed a vast network of trade and exchange that facilitated the movement of commodities and agricultural products across medieval Central Asia via settled urban communities and mobile pastoralists. Considering food consumption patterns as an expression of socio-economic interaction, we analyse human remains for carbon and nitrogen isotopes in order to establish dietary intake, then model isotopic niches to characterize dietary diversity and infer connectivity among communities of urbanites and nomadic pastoralists. The combination of low isotopic variation visible within urban groups with isotopic distinction between urban communities irrespective of local environmental conditions strongly suggests localized food production systems provided primary subsistence rather than agricultural goods exchanged along trade routes. Nomadic communities, in contrast, experienced higher dietary diversity reflecting engagements with a wide assortment of foodstuffs typical for mobile communities. These data indicate tightly bound social connectivity in urban centres pointedly funnelled local food products and homogenized dietary intake within settled communities, whereas open and opportunistic systems of food production and circulation were possible through more mobile lifeways.

## Introduction

Medieval Central Asia (ca. AD 2^nd^–16^th^ c.) was a locus of extensive cultural and economic interaction between East Asia, the Middle East, and Europe through a vast network of overland trade routes, commonly called the ‘Silk Roads’^1–4^. Urban centers, located in fertile oases and often described by archaeologists and historians alike as cosmopolitan cities^5,6^, helped anchor Silk Road exchange and foster early globalization across Asia^7,8^. Although settled communities provided a substantial economic foundation through agricultural output and the manufacture of valuable craft commodities such as metals, ceramics, glass, and textiles^9–14^, mobile pastoralists also had strong influence on the trade system as operators of highland pathways that were based on seasonal movements for herding livestock^15^.

While transfers of objects and materials have long represented the intensity and scope of Silk Road exchange^16^, we still lack detailed data about the way food systems, which reflect sustained engagements with the environment and broader community dynamics, were influenced by these far-reaching economic networks. Medieval Central Asia was defined by unusually diverse multicultural intersections, sudden social upheavals, and frequent demographic movements (Supplementary Information 1), but fundamental and perhaps durable dealings with food remain unclear. Food is culturally expressive of ecological adaptation, social relations, ideology, and economy^17–21^. Resolving dietary diversity in ancient Central Asian foodways provides an opportunity to investigate subsistence models of ‘nomadic’ and ‘urban’ communities that, together, played key roles in transcontinental interaction across the Silk Roads.

In Central Asia, which is characterized by strong seasonal climate and uneven distribution of resources on the landscape^22,23^, there is high potential for dietary diversity among pastoralist communities. Mobile pastoralists heavily subsist on livestock herding but also draw from a variety of food resources beyond domesticated animal products, including cultivated cereals, wild plants, fishes, and hunted game, which are exploited and consumed at varying intensities depending on environment, seasonal availability, mobility, and social networks^24–28^. On the other hand, medieval urban food systems were strongly invested in cereal agriculture, food storage, and sedentism^29,30^, and were subject to powerful political and religious institutions^7^, which may have generated less diverse dietary repertoires. However, dynamic commercial, political, and social activities between population centers and peripheral settlements, in addition to transactions with pastoralists, could have greatly expanded food availability and choice, as people and provisions, such as grains and live animals, regularly moved between urban and nomadic domains^31–35^.

We consider community-level dietary breadth over long periods of human life history to be a marker of dietary connectivity, which represents the cultural integration of food production, distribution, and consumption among individuals. Through globalization processes, which involve growing economic networks between increasingly distant communities, cultural differences diminish as groups cooperate and synchronize their consumption patterns, whether of foods, styles, or ideas^36^. In contemporary societies, globalized food economies expand with production standardization and increased dietary uniformity^37,38^. Along these lines, high intra-community dietary variability indicates that community members maintained divergent connections to food resources that express individual dietary preferences and group partitioning. Conversely, low dietary variability within communities signals converged trajectories of foods that reflect shared dietary practices and socio-economic coordination.

In order to establish diversity in human dietary intake in Central Asian urban and nomadic communities, we analysed the carbon and nitrogen stable isotopic composition of bone collagen from human remains of 74 individuals (Table 1). Carbon and nitrogen stable isotope ratios (δ^13^C and δ^15^N) provide an integrative measure of dietary intake^39,40^, and human bone collagen reflects a 10–15 year rolling average of protein consumption^41–43^. We model isotopic niches in bi-variate isotopic space (δ-space) to estimate the breadth and structure of community-level diets based on intra-group variation across both dimensions of isotopic ratios simultaneously^44,45^. Food resources with distinct isotopic content, which are incorporated into individual diets in various proportions, drive community dietary breadth^46^. The isotopic niche modelling uses Bayesian inference to fit standard ellipses to data points that are then expressed as probability distributions of area (‰^2^) and position in δ-space^47^. Crucially, we performed redundancy analysis to unravel isotopic diversity driven by culturally defined food choices from environmental variables that influence the isotopic composition of food resources.

**Table 1:** Geographic and archaeological information about analysed sites, including number of human samples. Detailed archaeological information for each site is provided in Supplementary Information 3. *See methods.

Country	Region	Site	Elevation (m.a.s.l.)	Chronology	n	Archaeological context	References
Uzbekistan	West Pamir-Alay	Tashbulak	2100	9th–11th c.	4	Highland urban complex of the Qarakhanid Empire; citadel, metal workshops, necropolis; 7 ha	^86,120^
		Alyntepe	475	10th–13th c.	1	Provincial city with fortified walls and surrounding settlements; industrial scale brick and ceramic production; 40 ha	^121^
		Frinkent	530	10th–13th c.	4	Fortress complex with cemetery of unique Zoroastrian burials in large ceramic vessels; 14 ha	^122,123^
	Ferghana Valley	Chor Dona	580	11th–13th c.	4	Fortress mound with associated grain processing facility and ancillary ancient settlement of Andijan; estimated 10–15 ha	^124,125^
		Chartok	545	12th c.	11	Context information is not available	^126^
	Tashkent Oasis	Uturlik	270	12th c.	9	Large city with diverse economic production of crafts; along trade routes with Otrar; 60 ha	^34,127^
	Khoresm	Tok-kala	60	9th–12th c.	9	Urban fortress and surrounding settlements that functioned as a regional centre of political and economic influence; estimated 10–15 ha	^128^
Kazakhstan	Otrar Oasis	Konyr-tobe I	180	5th–7th c.	9	Cemetery platform raised 2.5 m above ground level on the outskirts of a fortress; burials suggest nomadic traditions	^81,129^
		Temirlanovka	320	2nd–4th c.	4	Cemetery unassociated with a settlement containing burials of nomadic individuals	SI 3
	Zhetysu (Semirech’ye)	Turgen II	1040	2nd–6th c.	7	Settlement and burial complex with nomadic occupations from the late Bronze Age to medieval period; estimated < 5 ha	^79^
		Butakty II	1150	10th–12th c.	6	Settlement and burial complex with nomadic occupations from the late Bronze Age to medieval period; estimated < 5 ha	^80,130,131^
		Karatal	620	8th–11th c.	3	Cemetery complex in use from the late Bronze Age to historical period; numerous nomadic encampment structures	^84^; SI 3
Turkmenistan	Dehistan Plain	Geotchik Depe	130	Iron Age*	2	Urban complex with occupation from the early Iron Age to late Islamic period; 5.5 ha	^102,132,133^
		Misrijan	170	11th–12th c.	1	One of numerous large villages in the Misrijan Oasis; precise site is not reported in the literature	^102,132,133^

### Isotopic variation in Central Asia

**Figure 1:**
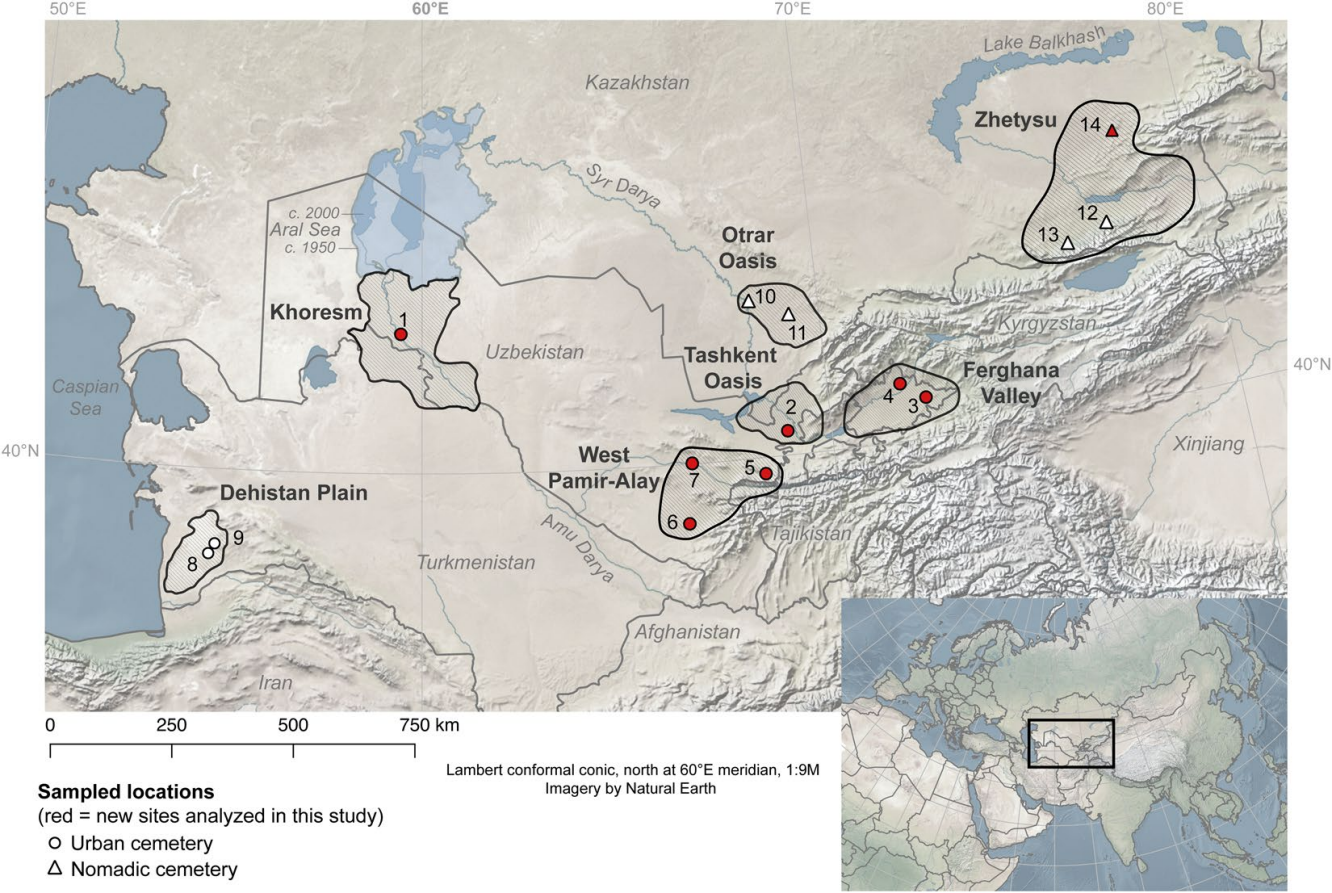
Map of Central Asia showing sites and regions with human stable isotopic data (δ^13^C and δ^15^N) analysed in this paper. Uzbekistan: 1) Tok-kala, 2) Uturlik, 3) Chor Dona, 4) Chartok, 5) Tashbulak, 6) Altyntepe, 7) Frinkent; Turkmenistan: 8) Geoktchik Depe, 9) Misrijan; Kazakhstan: 10) Konyr-Tobe, 11) Temirlanovka, 12) Turgen, 13) Butakty, 14) Karatal. Map generated with Quantum GIS, version 2.18.2 (https://www.qgis.org), using public domain data from Natural Earth (http://www.naturalearthdata.com).

We sampled 14 cemeteries associated with medieval Silk Road communities that span a long transect of Central Asian geography to include present-day Kazakhstan, Uzbekistan, and Turkmenistan (Fig. 1). The human individuals analysed in this study represent urban and non-urban consumers, who potentially had access to substantial food options that were available through prolific agricultural systems and marketplaces that drew in people and foodstuffs from oasis, desert, steppe, and highland environments (Supplementary Information 2). Diverse food remains were recovered from the sites represented in this study, which included cereals, legumes, fruits, fish, and livestock (Supplementary Information 3).

Pronounced variation in the regional environments and topographies, combined with strong seasonality, in which these sites are situated, confers high isotopic variation in Central Asian foodwebs accessed by people. Vegetation communities range from cool montane meadows and forests consisting of largely C_3_ taxa to hot lowland deserts that support both C_3_ and C_4_ plants^48–51^, which exhibit (pre-modern^52,53^) δ^13^C values of −25 ± 2–5‰ and −11 ± 1‰, respectively^54–57^. Food crops exploited in medieval Central Asia included the full spectrum of Eurasian domesticates including C_3_ taxa, such as wheat, barley, rice, nuts, and fruits, and C_4_ taxa, such as millets^58^. Although nitrogen isotopic variation in vegetation communities in Central Asia is under-characterized, research in ecosystems comparable to those of Central Asia, such as the Gobi steppe-desert^59^, the steppe deserts of the Caspian Depression^60^, and semi-arid western Loess Plateau^61^, demonstrate wide variation in plant δ^15^N values, ranging from −5 to 14‰, due to differences in local soil nitrogen pools and animal stocking rates^62–65^. In general, fish exhibit high δ^15^N values relative to terrestrial fauna^66,67^, and in Central Asia, fish exhibit an apparent continuum of δ^13^C values from ca. −11.5‰ to −27‰^68^.

## Results

**Figure 2:**
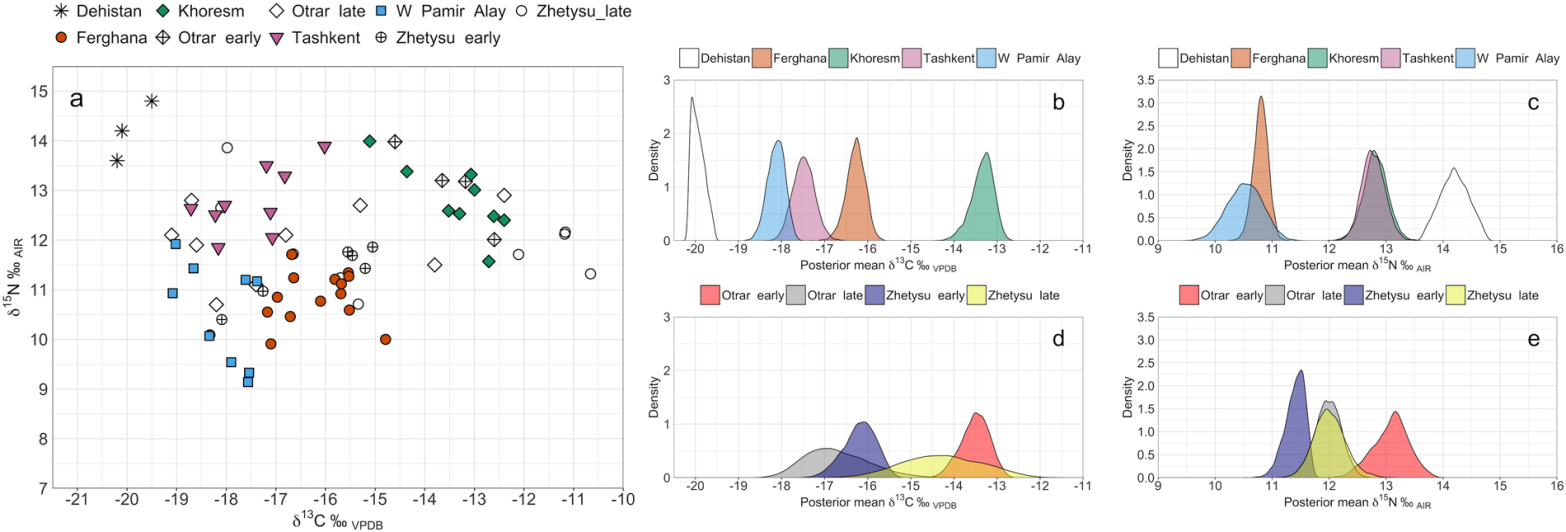
(**a**) Human carbon and nitrogen isotopic ratios from medieval Central Asia; (**b–c**) Posterior probability distributions of isotopic means obtained by Bayesian bootstrapping (mean_b_) from medieval urban communities in Uzbekistan and Turkmenistan and (**d**-**e**) from medieval nomadic communities in southern Kazakhstan.

Human remains in this study represent two chronological intervals of the medieval period. The bulk of the dataset (n = 63) dates to a ‘mid-late’ period of 6^th^–13^th^ c., which were recovered from sites in southern Kazakhstan, Uzbekistan, and western Turkmenistan. A small sample (n = 11) dates to an ‘early’ period of nomadic occupation at sites in southern Kazakhstan, which allows for a diachronic comparison of nomadic dietary intake within this region. Overall, human isotopic values range from ca. −20‰ to −10.5‰ for δ^13^C and 9‰ to 15‰ for δ^15^N (Fig. 2a). Isotope values cluster in both δ^13^C and δ^15^N for urban communities on a regional basis, while pastoralist communities from later periods exhibit wide distributions of δ^13^C values and relatively narrow ranges of δ^15^N values. Subsequent analyses using Bayesian inference clarified isotopic differences among communities while factoring in uncertainty due to small sample sizes. Summary statistics of isotopic data are provided in Supplementary Figure S1 and Table S1; raw isotopic data are provided in Supplementary Tables S2–S4.

### Human isotopic variation across medieval Central Asia

Significant distinctions in the dietary intake of medieval urban and nomadic communities across Central Asia are revealed by Bayesian means (mean_b_) of δ^13^C and δ^15^N values. Sharp dissimilarities are observed between urban communities located south of the Syr-Darya river, which provides a rough environmental boundary between the semi-arid/mountain steppe landscapes in southern Kazakhstan and sandy desert landscapes interspersed with oases and foothill zones to the east. Significant differences in the 95% CIs of isotopic values from each southerly community, with the exception of West Pamir-Alay and Tashkent, are present with mean_b_ δ^13^C values of −19.9‰ for Dehistan, −18.1‰ for West Pamir-Alay, −17.5‰ for Tashkent, −16.3‰ for Ferghana, and −13.3‰ for Khoresm (Fig. 2b; Information 4). Regional communities in Uzbekistan and Turkmenistan fall into in three trophic groups, each separated by ca. 2‰ in mean_b_ δ^15^N (Fig. 2c).

A diachronic shift in the dietary intake of nomadic communities located in southern Kazakhstan is indicated by a significant 3‰ decrease in mean_b_ δ^13^C values between early and late Otrar (Fig. 2d; Supplementary Information 4) and an increase of ca. 2‰ in mean_b_ δ^13^C between early and late Zhetysu. However, mean_b_ δ^13^C for late Zhetysu has a wide 95% CI between −15.9 and −12.3‰, which precludes a reliable estimate of the change. Overall, mean_b_ δ^15^N for communities in southern Kazakhstan are more mutually similar to each other than that in southerly regions (Fig. 2c,e). Early Otrar displays mean_b_ δ^15^N that is spaced apart by slightly less than ca. 1.7‰ from that of early Zhetysu, while in the later period the regions have identical mean_b_ δ^15^N of ca 12‰.

### Environment and isotopic variation

Isotopic patterns observed at individual archaeological sites are not driven by environmental inputs. Redundancy analysis between mean δ^13^C and δ^15^N values per cemetery site and 25 environmental parameters of ecologically relevant rainfall and temperature variations, in addition to elevation and soil properties, did not result in statistically significant relationships. Multiple linear regressions were also performed, which further failed to generate statistically significant relationships. (See Supplementary Information 5 for methods and results.)

### Isotopic niche modelling

**Figure 3:**
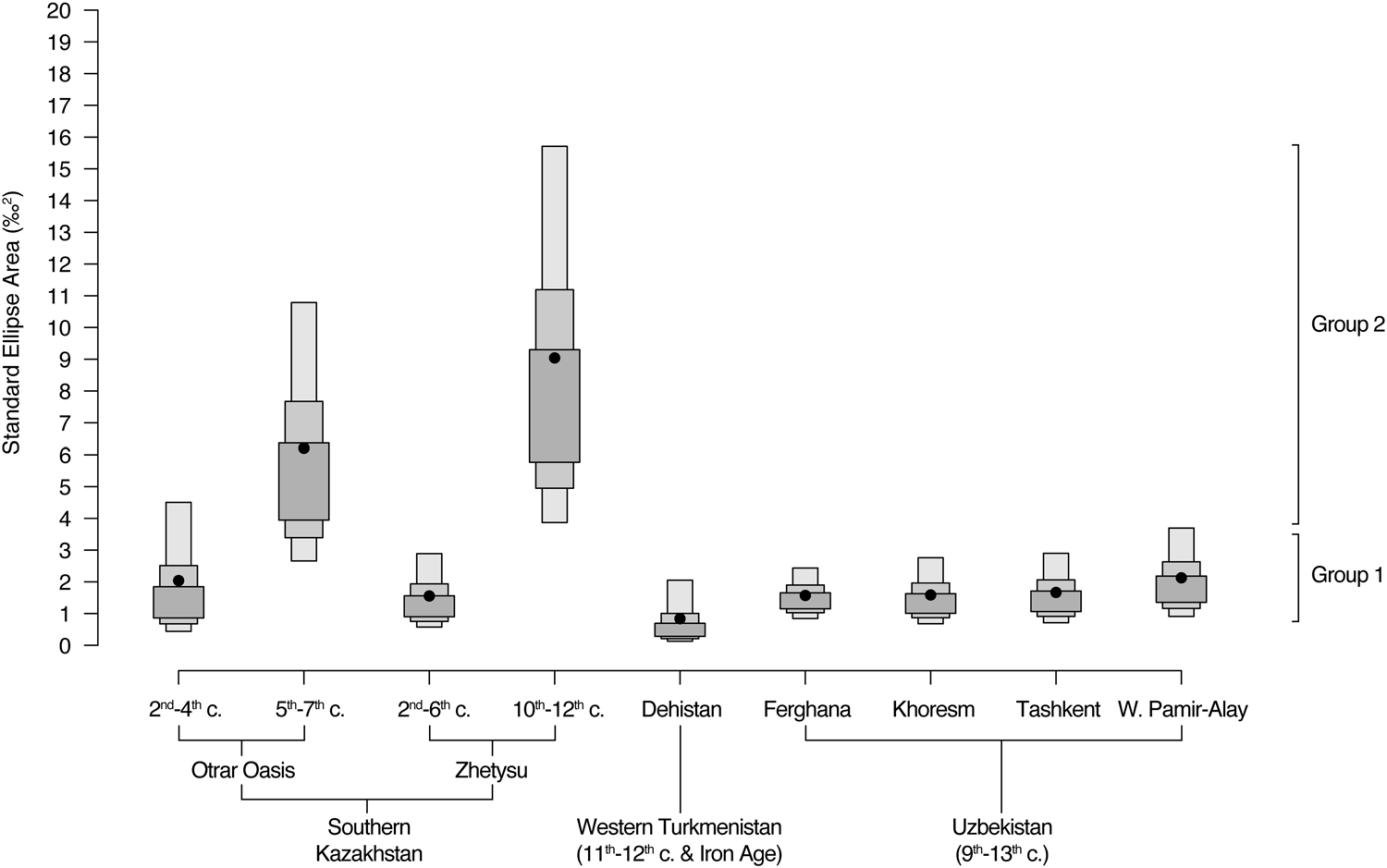
Community-level dietary diversity of medieval humans represented by posterior distributions of core isotopic niche area (‰^2^) by sites and regions in Central Asia. Isotopic niches were calculated by fitting standard ellipses to cover ca. 39% of the δ^13^C and δ^15^N data points using Bayesian inference. Black dots indicate area means, and the shaded boxes, from dark to light, represent the 50%, 75%, and 95% credible intervals.

Central Asian medieval communities display isotopic niches that cluster in two distinct size ranges (Fig. 3; Supplementary Information 6). Group 1 exhibits small areas between 0.1 and 3.7‰^2^ for urban communities in Uzbekistan and Turkmenistan, as do nomadic communities in southern Kazakhstan from the early medieval period. Over the next several hundred years, dietary diversity among individuals in Zhetysu and the Otrar Oasis (Group 2) appears to have radically increased, with isotopic niches estimated between 2.7 and 15.7‰^2^. Notably, the greatest intra-community dietary diversity is visible in late Zhetysu, indicated by a 95% CI exceeding that from Group 1 communities, except early Otrar, which slightly overlaps by 0.7‰^2^.

**Figure 4:**
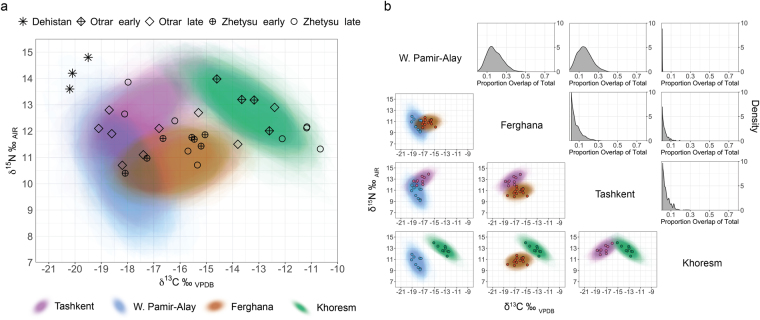
(**a**) Medieval urban isotopic niches from Uzbekistan are displayed as probability clouds, and individual isotopic values from southern Kazakhstan (nomadic communities) and western Turkmenistan (urban community) are represented as points. (**b**) Isotopic niche overlap analysis for urban communities in Uzbekistan. Standard ellipses covering 95% of δ^13^C and δ^15^N values were modelled using Bayesian inference. Overlapping areas for each pairwise comparisons in δ-space were visualized as probability clouds with underlying isotopic data points superimposed (lower left). Area overlap of total isotopic niche area for each pair was plotted as probability distributions (upper right).

While all narrow, modelled isotopic niches for medieval urbanites in Uzbekistan are highly unique in orientation and position in δ-space (Fig. 4a). For late Zhetysu and Otrar, the diffuse spacing of isotopic values indicates exceedingly varied dietary intake. Individuals in late Zhetysu, which represent two archaeological sites, fall within the isotopic niches for Khoresm, Ferghana and Tashkent. Likewise, individuals in late Otrar, which represent one site, span all four urban isotopic niches from Uzbekistan. In contrast, there is substantial proportional overlap between the small isotopic niches from early Zhetysu and Ferghana and also early Otrar and Khoresm, respectively (95% CI: 0.13–0.64 and 0.17–0.69; Supplementary Fig. S6), which indicates a high likelihood of dietary parity.

Isotopic niches from urban communities in Uzbekistan illustrate strong dissimilarity from each other (Fig. 4b). The highest proportion of niche overlap occurs between West Pamir-Alay and Ferghana (95% CI: 0.05–0.36) and between West Pamir-Alay and Tashkent (95% CI: 0.01–0.32), suggesting that a maximum of one-third of individuals in these communities had similar dietary intake. However, this niche overlap could be as low as 1–5%. The remaining pairwise comparisons of urban communities show negligible occurrence of overlap (Supplementary Information 7).

## Discussion

Taken together, urban and nomadic communities display distinctive but wide-ranging isotopic values that are strongly suggestive of diverse dietary intake across medieval Central Asia. A large overall range of human δ^13^C values from ca. −20 to −11‰ is likely due to individuals consuming mostly C_3_ crops (wheat, barley, and rice) or C_4_ millets at sustained intensities. Among Eurasian cultigens, millets are isotopically distinct with high ^13^C concentration. Millets thrive in hot and arid climate and exhibit fast-growing and drought-tolerant adaptations^69^, traits which would have provided Central Asian farmers and mobile pastoralists opportunities for low risk, low-investment cultivation in marginal agricultural areas^70,71^. The natural abundance of C_4_ vegetation is substantially higher in the desert zones of Central Asia^50^, where livestock, as part of urban or nomadic subsistence, could have accessed enriched δ^13^C biomass and provided human consumers with protein-dense foods (meat and milk) with high δ^13^C values. A lack of correlations between human isotopic values and site environmental parameters suggests that dietary intake across the region was shaped primarily by food choice. Medieval agriculture in Central Asia, which used complex crop schedules and large irrigation works^71–76^, likely enabled productivity to overcome environmental constraints on crop variety in order to meet the inter-connected dietary demands of consumers.

Modelled isotopic niches indicate that nomadic communities exploited a wide variety of dietary resources, while urban communities engaged in more limited dietary repertoires. The small isotopic niche sizes documented among urban communities in Uzbekistan and Turkmenistan suggest food channels that were shaped via consistent and insular dietary connectivity. On the other hand, large isotopic niches in southern Kazakhstan from a relatively contemporaneous period indicate that a multitude of food acquisition strategies were in use, by which communities tied more closely with pastoral nomadic lifeways comprised individuals with assorted dietary relationships that led to sustained differences in isotopic variability. The dietary connectivity for these nomadic groups may have fostered group partitioning through unsynchronized food interactions among different community members. The small isotopic niches for nomadic communities in early Otrar and Zhetysu, which in this case substantially overlap with that for Khoresm and Ferghana, suggesting more restricted dietary intake by pastoralists, emphasize the subsistence plasticity of pastoral nomads who readily contour their own food production and interaction networks in response to dynamic social and natural landscapes^24,26,77^.

The diachronic shift in isotopic niche size between early and late nomadic communities also highlights two distinct scales of dietary variability that illustrate the importance of multi-resource pastoralism to Silk Road interactions. In southern Kazakhstan, the early medieval period is marked by a growth of urban centres, villages, and agricultural economies^78–81^, which is also historically associated with frequent conflict among nomadic confederacies that instigated socio-political turmoil^1,82^. In order to mitigate risk and take advantage of economic opportunities presented by these newly founded centres, nomadic communities likely participated in coordinated subsistence interactions with settled populations over short distances, which would have effectively limited access to diverse food resources and thus narrowed their dietary breadth. During the strengthening of Turkic empires several centuries later^1,3,31,83^, Silk Road trans-regional trading expanded to include bulk commodities and raw materials^32^, and nomadic communities across southern Kazakhstan expressed wide dietary breadth, as indicated by large isotopic niche sizes.

One explanation for greater inter-individual dietary diversity during this later medieval period is that nomadic communities tapped into growing trade economies as agents of food exchange and broke out of insular urban subsistence channels. Recent excavations of nomadic encampments in the foothill zones of West Pamir-Alay and Zhetysu illustrate highly variable levels of economic interaction between pastoralists and urban centres. At these sites, cultural materials associated with 8^th^–13^th^ c. radiocarbon chronologies include cotton fabrics^84^ and variable mixtures of ceramics ranging between standardized wheel-spun food storage vessels from distant oases communities and locally produced ‘handmade’ coarsewares, which are rare in urban contexts^85,86^. The presence of hybrid ceramic assemblages and cotton, a woven trade good associated with oasis production centers^87,88^, suggests that complex and non-uniform relationships with urban economies coincided with intra-group dietary diversity in mobile pastoralist communities^25^.

Accordingly, at the community level, a second scale of dietary variability that is representative of multi-resource pastoralism is observed at late Otrar and Zhetysu, which display wide ranges of δ^13^C values (Fig. 4a). Some individuals in late Zhetysu have δ^13^C values similar to those commonly observed in humans from prehistoric millet-based farming societies in China, where millets were domesticated^89–91^, while individuals in late Zhetysu and Otrar had low δ^13^C values, typical of Neolithic and early Bronze Age humans before millet spread to the C_3_-dominant Central Asian steppe^92^. Both communities in late Otrar and Zhetysu display similar δ^15^N distributions, though individuals exhibit differences in dietary intake of relative proportions of meat and dairy products (Fig. 2e). Together, these findings suggest that dietary connectivity at the steppe margins was associated with an ecumene of diverse food consumption, in which individuals maintained separate subsistence strategies as they simultaneously participated in a common nomadic ethos. Compared to urbanites, mobile pastoralists likely maintained closer control of food production and distribution, allowing them to eat according to food preferences, which may have been less important for maintaining social ties than in urban contexts.

The combination of narrow dietary niches with isotopic distinction in human remains from medieval urban communities south of the Syr-Darya river is due to trophic-level variation in the intensity of meat and cereal consumption between communities as well as differences in the contribution of millet to urban diets, either eaten directly or indirectly obtained from meat and dairy of animals foddered with millet. Isotopic niche overlap was highest for urban communities in close geographic proximity to one another, a pattern that suggests neighbouring communities either participated in similar food traditions or agricultural practices that were confined to small catchments. These communities may have participated in limited inter-regional trading of staple foods, which likely would have moved through established subsistence channels as if locally produced. There is also the possibility that dietary connectivity in urban contexts was subject to bureaucratic intermediaries, which exerted influence through land tenure and taxation^1,32,93^. Alternatively, in the absence of top-down control, food exchange networks may have steadily channelled provisions to urbanites as a reflection of other economic networks that inevitably developed to be streamlined towards cultural insiders in cosmopolitan contexts.

While the consensus among historians and archaeologists is that urbanites in medieval Central Asia dwelled in rich multicultural settings^5,7,16^, there appears to be a limit to this diversity in dietary intake as revealed through isotopic niche modelling. Distinctions in food choice and diet between urban communities suggest regional food repertoires were narrowly circumscribed, at least between C_3_ and C_4_ crops and also between animal and plant protein. Regional patterns in diet imply that cultural differences surrounding foods may have been surprisingly diminished within urban communities, which runs counter to the notion of collective cosmopolitanism in medieval Central Asia. Medieval urbanites in Central Asia maintained inward-focused dietary connectivity that likely generated a localized social cohesion through culturally integrated supply chains for consumers.

Scholars also associate Silk Road activity with early globalization processes^7,8^, in which urban centres are viewed as the main drivers of cultural influence and outward economic connectivity, while ‘nomads’ are interpreted as antagonists to ancient civilization^94–96^. Yet, through multi-resource subsistence strategies, nomadic communities likely wielded flexible economic engagements that traversed open landscapes of contact with people who facilitated far-reaching connectivity. In this sense, nomadic individuals may have been more culturally interoperable and able to participate in, disengage from, and influence cultural spheres more easily than urban populations. Indeed, many of the pan-regional turnovers in religion, language, and political authority that resulted in changes in architecture, technologies, and other commodity classes in the medieval period are historically described as nomadic innovations^1,2,31^, and essential routes that connected Silk Road sites in the highland regions of Central Asia were likely shaped by nomadic mobility^15^. This study takes a new step toward resolving the complex interplay between urban and nomadic societies that are rarely available through archaeological datasets. Establishing dietary diversity provides an emerging understanding of food and connectivity along Central Asia’s Silk Roads that highlights the significance of ancient nomadic pastoralists in bridging seemingly insulated urban centres.

## Materials and Methods

### Human remains

We performed new analyses on human remains from Uzbekistan and Kazakhstan, which were selected based on 1) medieval chronology from ca. 8^th^–13^th^ c., 2) information on archaeological context, and 3) minimum age estimation of young adult. The majority of human remains analysed from Uzbekistan (n = 38) were excavated at various times over the past 80 years and do not include associated post-cranial elements. Human remains from Tashbulak (n = 4) were excavated in 2015 and are represented by complete inventories of skeletal elements. Human remains from Uzbekistan are stored in the Institute of Archaeology of the Uzbek Academy of Sciences in Samarkand, under the auspices of the Archaeology of the Qarakhanids Project (Co-PIs: Farhod Maksudov and Michael Frachetti). Human remains from Karatal (n = 3) were excavated in 2006 and 2008 and include incomplete skeletal elements due to ancient burial looting and modern erosion (Supplementary Information 3). Human remains from Karatal are stored at the Central State Museum of Kazakhstan in Almaty, under the auspices of the Dzhungar Mountain Archaeology Project (Co-PIs: Alexei Mar’yahshev and Michael Frachetti). A list of human samples is provided in Supplementary Tables S2–S4.

### Isotopic analysis

Approximately 1–2 cm^3^ of sample were cut from dense cortical bone. Collagen extraction was performed in the Archaeological Stable Isotope Laboratory of Kiel University following Tuross *et al*.^97^. Mass spectrometry for δ^13^C and δ^15^N was performed at the Boston University Stable Isotope Laboratory using a EuroVector Euro EA elemental analyser coupled with a GVI IsoPrime in continuous flow mode with an analytical error of 0.1‰ and 0.2‰ for δ^13^C and δ^15^N, respectively. Isotopic values are reported in permil (‰) relative to the Vienna Pee Dee Belemnite (VPDB) standard for δ^13^C and atmospheric nitrogen (AIR) for δ^15^N. Collagen samples with an elemental C:N ratio less than 2.9 or greater than 3.6 were considered diagenically altered and unsuitable for inclusion^98–100^. Failed samples (n = 3) are reported in Supplementary Table S5.

Previously published δ^13^C and δ^15^N values of human bone collagen from southern Central Asia were analysed (Supplementary Tables S3-S4). Isotopic data from medieval southern Kazakhstan included 13 samples from the Otrar Oasis (3^rd^–7^th^ c.) and 13 samples from southern Zhetysu (2^nd^–12^th^ c.)^101^. Data from the Dehistan Plain in western Turkmenistan included two samples from the Iron Age (ca. 1300 BC) and one sample from the medieval period (ca. 11^th^–12^th^ c.)^102^. These data were lumped together irrespective of chronology due to low sample size for the region and similar isotopic values, which were unique in our dataset.

Human collagen is δ^13^C enriched by ca. 1–3‰ relative to the carbon isotope composition of consumed foods^103^, whereas herbivores are enriched in δ^13^C by ca. 5‰ relative to consumed vegetation^104,105^. Human nitrogen isotope values reflect the intensity of cereal versus meat consumption, as there is a 2–5‰ trophic enrichment in δ^15^N with each step up in the food web^40,106,107^, but also reflect variation in food production systems that introduce exogenous nitrogen, usually in the form of manure, to the floral base of the food web, potentially imparting considerable nitrogen isotopic variation in agricultural and livestock food^62,63,65,108,109^. In attempts to estimate the relative contribution of specific foods in diets, a common approach in archaeological dietary studies is to sample ancient and modern plants and animals to assess possible isotopic variability in food sources that result from various environmental and anthropogenic factors^110^. For the purposes of establishing human dietary diversity at the community level, however, knowledge of the isotopic content of ancient food remains is unnecessary.

### Statistical analysis

All statistical analyses were performed using R, version 3.4.0^111^. Due to low sample sizes per site (1 ≤ n ≤ 11), δ^13^C and δ^15^N values were analysed by geographic region (3 ≤ n ≤ 15) using Bayesian techniques to quantify uncertainty and overcome issues with non-parametric data distributions, which cannot be reliably analysed with frequentist methods. The means of isotopic values for each region were calculated with a Bayesian bootstrapping method at 5000 iterations using the package bayesboot^112^. Posterior distributions of means for each group were considered statistically different from one another if the 95% credible intervals (CI) did not overlap.

### Isotopic niche modelling

Isotopic niches were analysed using SIBER (Stable Isotope Bayesian Ellipses in R), version 2.1.3^47^. The Markov chain Monte Carlo simulation was run with uniform priors 2,000,000 times, with the first 10,000 results discarded (burn-in), followed by a 1:100 thinning. The fitted ellipses express a posterior probability distribution of area (‰^2^) and position in δ-space. This technique is statistically advantageous for analysing communities of consumers, as fitted ellipses are less sensitive to sample size than other spatial metrics, such as convex hulls^47^, and uncertainty is factored into estimates, such as sample size, which has previously challenged studies using a finite number of specimens from the archaeological record. Isotopic niches were compared among regional groups by using the Bayesian standard ellipse areas (SEA_b_) and the proportion of SEA_b_ overlap as total SEA_b_ pairwise for each region. Overlapping areas of isotopic niches indicate similarity in isotopic inputs and thus comparable resource exploitation^113–115^.

### Environmental modelling and GIS

Interactive effects between human stable isotope values and environmental parameters at each site in 10 km and 50 km spatial buffers were explored using redundancy analysis (RDA) with the R package vegan, version 2.4–5^116^. Environmental parameters included elevation, bioclim data^117^, which are derived from monthly temperature and rainfall values from 1970–2000, and soil parameters obtained from the Soil Grid Project^118^. (See Supplementary Information 5 for RDA methods and results.) Data were mapped using Quantum GIS, version 2.18.2^119^. Base imagery in Fig. 1 was obtained from Natural Earth (http://www.naturalearthdata.com).

## Electronic supplementary material

Supplementary Information
