# Supplementary material for: Urban and nomadic isotopic niches reveal dietary connectivities along Central Asia’s Silk Roads
Source: Sci Rep. 2018 Mar 26;8:5177. doi: 10.1038/s41598-018-22995-2 (PMC5979964; doi:10.1038/s41598-018-22995-2)
Supplement: Supplementary file 1 — Supplementary Information [file 41598_2018_22995_MOESM1_ESM.pdf]

Supplementary Information

Urban and nomadic isotopic niches reveal dietary connectivities along Central Asia's Silk Roads

Taylor R. Hermes[1,2]*
Michael D. Frachetti[3]*
Elissa A. Bullion[3]
Farhod Maksudov[4]
Samiriddin Mustafakulov[5]
Cheryl A. Makarewicz[1,2]*

* Corresponding authors

Affiliations

1 Archaeological Stable Isotope Laboratory
Institute for Prehistoric and Protohistoric Archaeology
Christian-Albrechts-Universität zu Kiel
Johanna-Mestorf-Straße 2-6
24118 Kiel, Germany

2 Graduate School 'Human Development in Landscapes'
Christian-Albrechts-Universität zu Kiel,
Leibniz Straße 3
24118 Kiel, Germany
trhermes@gshdl.uni-kiel.de
c.makarewicz@ufg.uni-kiel.de

3 SAIE Laboratory
Department of Anthropology
Washington University in St. Louis
One Brookings Drive, CB 1114
St. Louis, MO 63130, USA
elissa.bullion@wustl.edu
frachetti@wustl.edu

4 Institute for Archaeological Research,
Academy of Sciences of Uzbekistan
Yahya Gulamov Street No. 70
Tashkent, 100000, Uzbekistan
fmaksudov@yahoo.com

5 Afrasiyab Museum
Islam Karimov Street No. 7
Samarkand, 114151, Uzbekistan
m_samar@rambler.ru

Supplementary Information 1: Culture history of increased interaction in medieval Central Asia

From the 2rd to 6th c., the earliest dates of our human samples, Central Asia was characterized by ethnically diverse and politically independent city-states, occasionally subjugated by distant powers, that maintained sustained interactions with Turkic nomadic groups located in the steppes of present-day Kazakhstan, Uzbekistan, and Xinjiang[1]. During the 7th-8th c., the Islamic political authority spread into the settled oases of the Silk Road system[1,2], which is associated with a strengthening of the social integration between institutions in urban economies[3], expansion of long-distance trade[4], and mutual commercial prospects for nomads and urbanites[2,5].

By the 9th-10th c., Central Asia was generally divided between the Samanids, an Islamic dynasty of Persian origin that controlled lowland oases, and Turkish-speaking nomadic hegemonies native to the steppe and highland zones[6,7]. In the 10th-11th c., the Qarakhanids, an imperial confederacy of nomadic lineages, conquered Central Asia and populated urban institutions with its nobility, which expanded urban projects and, supposedly, was followed by an influx of peasant nomads into cities and villages but a concurrent political expansion into highland zones[1,2,8,9]. The Qarakhanids continued to rule Central Asia until about the mid-12th c. when the empire fragmented[1,7], and the Mongols swept over the region in the early 13th c.[1,10], which marks the nearest historical boundary of our study.

Supplementary Information 2: Subsistence review for medieval Central Asia

The food production systems of medieval Central Asia were supported by sophisticated irrigation technology as well as extensive rainfed farming and livestock management that, together, generated a diverse and plentiful supply of crops and animal products to both urban and nomadic realms[11–15]. Ancient texts of Chinese and Arab geographers describe lush Central Asian oases where wheat, barley, millet, rice, peas, chickpeas, lentils, melons, and cotton were cultivated, in addition to gardens, vineyards, and orchards of fruit and nut trees[1,16,17]. Incidental recovery of carbonized botanical remains from archaeological sites and ubiquity of agricultural artefacts confirm these cultigens were grown and processed throughout the region[18–24]. More systematic recovery of botanical remains confirms a rich diversity of cultivated taxa with a strong emphasis on various grains as staple foods[25–29], alongside local pastoral production and, presumably, animal products acquired from local nomadic herders[18,30,31].

The historical record further documents bustling fairs and markets in which foods were traded in bulk and also exchanged in quantities for individual households or estates. The geographic extent of this commerce has been documented through ceramics artefacts, which derive from major urban production centers in Samarkand, Tashkent, Bukhara, Afghanistan, Iran, China and beyond[1,6,18,31]. One of the most prominent markets took place in the Zaamin Mountains., in a city called Marsmand, which was supposedly located next to a major centre for mining ore and metal production named Mink[18,19]. While existing textual accounts and subsistence datasets have provided insights into interactions with food in urban centers and fortified settlements, no published data exists to elaborate upon subsistence for nomadic pastoralists in later medieval times. Inconsistent chronologies, geographic coverage, and recording methods preclude using existing data to understand regional dietary diversity and food exchange tied to the historical record.

Supplementary Information 3: Sampled sites, archaeological context, and subsistence data

Uzbekistan

Khoresm

Tok-kala is located near the modern city Nukus in Khoresm. The fortress, settlement, and cemeteries that comprise the preserved site cover approximately eight hectares and are located on a raised hill above the floodplain of the Amu Darya. An ancient river channel runs along the base of the hill, and there is evidence of multiple flooding events throughout its history, which appear to have destroyed much of the settlement architecture. Flooding in Khoresm is periodic, which has catastrophic potential to alter the course of the Amu-Darya and affect the level of the Aral Sea, and the harnessing of these floodwaters is considered to be a critical component to the success of irrigation systems in Khoresm[10]. During periods of vast hydrological change, such as in the 4th c., settlement patterns were drastically altered and sites were abandoned[32].

Archaeological materials recovered at Tok-kala seem to indicate significant interaction with nomadic groups in the region[21]. Besides a modern Muslim cemetery, there are three known cemeteries at Tok-kala[21]. The first is an early medieval (7th-8th c.) naos with ossuary burials, consistent with Zoroastrian practices. Notably, this phase contains well-preserved pieces of wall paintings and inscriptions on ossuaries that have led to speculation about their cultural origins, either directly from Sassanid roots or from older heritages pre-dating the Sassanid period[33]. The other two burial grounds are middle medieval (9th-11th c.) Muslim cemeteries, identified from grave forms and body position of interred individuals. Samples in this study were recovered from individuals in Muslim burials. The first of these cemeteries is located on top of the earlier Zoroastrian burials. Despite its relatively small size, Tok-kala is sometimes known as a 'besh-kala,' or mother-city, of Khoresm, indicating past cultural significance. Tok-kala is considered to have been a regional centre of political and economic influence[21].

The most comprehensive information about Tok-kala can be found in Gudovka's monograph[21]. She reports the recovery of wheat, barley, and millets from multiple cultural layers, but these macrobotanical remains were not quantified. Rich livestock and fish bones were also reported but were not quantified.

Tashkent Oasis

Uturlik sits on the left bank of the Syr Darya River in the Sirdarya Region of modern Uzbekistan, near Tashkent. In the historical and archaeological literature, the ancient city and local region are often referred to as Chach[31,34]. The site area covers approximately 60 hectares, and was occupied from the 6th-12th c.[35], and our samples from the site date to the 10th-12th c. A large medieval Muslim cemetery of approximately 4,000 graves is located on the western side of the site. The large area of the settlement and cemetery at Uturlik have led researchers to estimate a total population of between 8 and 10 thousand people at its peak[35]. The city is described as having a sprawling residential and artisan sections that provided a diverse and productive craft economy, in addition to vibrant markets[31]. Despite the apparent size of Uturlik, there is a paucity of detailed information in published sources about the ancient city. Scholars highlight the location of the city as being on an important trade route

between Samarkand, to the south, and the Otrar Oasis, located in Kazakhstan, which has led to speculation about interactions between settled and nomadic populations[31].
There does not appear to be any quantified subsistence information available for Uturlik, but scholars describe the ancient city as being supplied by irrigated and rainfed fields, in addition to livestock herding in the local hilly landscape[31]. There is the possibility that fishing occurred in the marshes, irrigation canals, and Syr-Darya river[31], but there is no material evidence for fishing reported in the literature.

Ferghana Valley

Chor Dona is located in the southeastern region of the Ferghana Valley, on the northwestern outskirts of the city of Andijan. The site was occupied during two periods: 7th-8th c. and 14th-16th c.[36]. Between these two occupations, the site was used as a cemetery. The majority of burials at Chor Dona are typical of Muslim practice, but there are at least five individuals buried with objects and grave structures similar to pre-Islamic rituals common in medieval sites in Kazakhstan associated with nomadic groups[36]. Chor Dona's proximity to ancient Andijan would have likely put its residents within the economic and social sphere of this large urban centre.

Chor Dona appears to have served as a large-scale economic production centre for grains, as evidenced by flour milling facilities[36]. Macrobotanical remains of foods recovered from the site include walnuts, millet, barley, and wheat, in addition to an assortment of agricultural tools associated with cultivation, harvesting, and processing[36]. Furthermore, fruit seeds of grapes, cherry plums, peaches, and apricots were reported as ubiquitous in the site[36]. Cotton seeds were also recovered[36].

Chartok is not well described in the archaeological literature. The site was published in a craniometric study on medieval populations in Uzbekistan that briefly describes Chartok as dating to the 12th c. and located in the Namangan region of northern Ferghana[34]. Unfortunately, no cultural context is given about the site. Searches for additional information did not yield results.

West Pamir-Alay

Tashbulak is a recently discovered site located in the highlands of the Malguzar Mountains, close to the border with Tajikistan, at approximately 2100 meters above sea level[37]. It is the only known highland centre that was constructed and occupied during the Qarakhanid Empire. This region has historically been occupied by nomadic groups, but during the 9th-13th c., a series of small cities and large villages were constructed in the mountain zone[38]. Tashbulak consists of an elevated citadel above a lower town area with workshops and possible residencies[9]. The site also contains a large Muslim cemetery of approximately 400 burials[9]. The site spans from the 9th to early 12th c., which is currently dated according to coins and Qarakhanid ceramics[9]. Research at the site is ongoing, but the presence of iron objects, glazed and wheel made ceramics, and non-local fruits indicate the likelihood that Tashbulak served as an important market, trading post, and/or production centre between highland and lowland regions. Macrobotanical remains of grains recovered from the site include barley and wheat, and millets were not found[9]; detailed paleobotanical work is forthcoming by Robert Spengler.

Altyntepe is in the western part of the Kashgardarya Oasis, near the modern-day town of Karabag. Although it was founded in the 7th-8th c., the site reached its peak in the 10th-13th c. and covered about 40 hectares[39]. The city consisted of a fortified inner city with surrounding sprawling settlements, and a cemetery is located on the southwest border of the site[39]. The remains of several different craft workshops have been recovered, including industrial-scale brick and pottery production complexes[39]. Despite evidence of economic fluorescence, some researchers point to the relatively small defensive walls as an indication that Altyntepe was not as important or wealthy as other medieval cities, such as Penjikent[39].

Macrobotanical remains at Alyntepe include watermelon, muskmelon, and grape seeds[39]. Excavations recovered bulk remains of wheat, which were reported as club wheat (*Triticum compactum*)[39]. Barley was not reported from Altyntepe, but small quantities were found in nearby sites[39]. Animal bones present in ash deposits were found throughout the site[39]. While fish bones were not reported, numerous ceramic bowls and dishes were recovered that were decorated with stamp impressions depicting slender symbols of fish[39].

Frinkent, also known as Afarinkend, is located in the lowlands of the Zerafshan valley, in the interfluve between the Kara-Darya and Ak-Darya rivers. Frinkent is sixty kilometres northwest of the city of Samarkand. The site, delineated by a wall, includes a fortress complex, cemetery, and settlement area covering approximately 14 hectares[40]. The site was founded in the 7th century by the brother of the ruler of Samarkand, making it a site of potential political importance[40]. The site continued to be an important centre of the Zoroastrian faith in the Zerafshan region even through the 10th century, when Islam had replaced most other faiths[41]. The cemetery at Frinkent is a stark example of this role. Hundreds of individuals were cremated or interred in large ceramic jars, some of which were painted on the exterior and had soot deposits as would have been caused by burning[40]. Many of the jars contained the skeletal remains of multiple individuals, and often the mandibles were missing, indicating skeletonization before primary or secondary deposition in the vessels[40]. Subsistence data could not be located for Frinkent.

Kazakhstan

Otrar Oasis

Konyr-tobe, also known as Kuyuk-Mardan, is a medieval fortress located on the Arys River. Two necropolises remain the only components of the site that have been systematically researched and published[42]. Konyr-tobe I contains 120 burials that were placed on a unique platform mound constructed from local clay-rich soil and mudbricks, which stands about 2.5 m above the ground level[42]. The platforms is outside of the fortress, and its association to the fortress is unclear. The human remains with stable isotopic data included in our study are sourced from these burials[43]. Located about 40 m from Konyr-tobe II is a similar but smaller platform mound with 8 burials[42]. Regional specialists consider these platform cemeteries, which are widely distributed in the Arys valley and Otrar Oasis, to have been built in order to stand seasonal floods in the area that heavily saturate the takyr surface[44]. This style of cemetery construction is unique to this site and scholars have interpreted it to represent nomadic communities[42].

Konyr-tobe I was in use from the 5th-7th c. based on iron weapons and implements, in addition to ceramic vessels, interred with the bodies[42,44]. Burial practices show resemblance

to pre-Islamic and late medieval rituals, despite artefacts largely reflecting an early medieval chronology[42,44]. This burial variability suggests high levels of interaction with outlying communities, which is further demonstrated by distinctive ceramic vessels with spouts common in the Konyr-tobe assemblage that were also found nearly 800 km away in contemporaneous layers of a nomadic encampment site in the desert-steppe margin of the Dzhungar Mountains, named Mukri[45]. In rooms of a nearby city, Kok-mardan, ceramic storage vessels were found with millet, wheat, barley, rice, and peas[42], but subsistence data for Konyr-tobe or its cemeteries were not reported.

Temirlanovka is an early medieval burial site located on the northern bank of Arys River. The site contains 49 burial mounds that date to the 2nd-4th c. based on material inventories of the graves[46] and one individual that was directly radiocarbon dated (cal AD 140-376)[43]. Most of the site remains unpublished, but an iron dagger from burial #33 has raised speculation that the community using the Temirlanovka burial ground had contacts with nomadic groups as far away as the Black Sea region, which during the late Iron Age can be generally described as a territory of the Scythian-Sarmatian cultural groups[47]. Pollen analysis from sediments recovered from ceramic vessels found in the burials revealed natural background vegetation of Ephedra and Artemisia, in addition to a small number of pollen grains from buckwheat and legumes[46].

Zhetysu (Semirech'ye)

Turgen is a multi-period settlement and burial complex in the modern city Turgen. The site contains mostly late Andronovo to Wusun cultural phases, but there is are also early medieval burials, which further extend across the entire floodplain the Ili Valley[48]. The burial forms include low earthen mounds surrounded by stone fences, which are characteristic of the Turkic period[48]. The subsistence economy of the early medieval period is believed to be similar to that of the late Iron Age, which included dense villages, irrigation agriculture, and management of livestock[49]. However, detailed excavation data for the site is not well reported. The chronology of the medieval burials is estimated to span the 2nd-6th c., according to burial forms and material remains that indicate nomadic traditions.

Butakty is a multi-period settlement and burial complex in the modern city Almaty. The site includes occupations from the late Bronze Age Andronovo cultural horizon (ca. 1700-1200 BC) to the medieval period (ca. AD 12th c.)[50,51]. The settlement areas contain rich artefact assemblages and architectural remains that indicate intensive, perhaps year-round occupation at the site[50,51]. The medieval burials are reported to be low in number, but they contain evidence of ritual practices common in the Zhetysu region, which includes burying sheep/goat limbs with the human bodies[51]. One individual from Butakty was directly radiocarbon dated to cal AD 1022-1155[43]. Iron fasteners and ring pendants share style with those characteristic in nomadic groups in central Kazakhstan and the Altai region[50,51]. No subsistence data could be located for Butakty, except brief mentions of an agro-pastoral economy that persisted throughout the site's occupational sequence[50,51]. (Butakty had been previously published with the incorrect geographical coordinates that placed it 10 km east of Turgen[43], but its location has been corrected in our paper, as shown in main text Fig. 1 and Table 1.)

Karatal is a multi-period, ancient cemetery complex located on the foothills of the Dzhungar Mountains and is about 1 km from the Karatal River. The site underwent partial excavation and survey in 2006 by Alexei Mar'yashev, Taylor Hermes, and Marc Meyer, and three

periods of use were identified (unpublished report). In 2008, another campaign continued work on medieval burials. The first occupation occurred during the late Bronze Age (ca. 1700-1200 BC), as indicated by distinctive stone burial cists that contained cremated human remains and 'Andronovo'-style ceramics. Some of the burial cists were arranged in pairs and enclosed within a low-profile stone fence. The next occupation occurred during the Iron Age (ca. 1000 BC – AD 300), as indicated by linear rows of earthen mounds (kurgans) placed on the flat areas of rolling hills. These kurgans were not excavated. Within the site's environs, numerous rock outcrops were found to be decorated with panels of Bronze and Iron Age petroglyphs that depict animals and anthropomorphic figures, which are common in the Zhetysu region[52,53]. Abandoned settlements, consisting of stone foundations for small structures (likely felt tents), were recorded on survey at low density, which suggests local residence by pastoralist groups.

The final phase of the Karatal cemetery complex occurred during the medieval period. Four adjacent burials containing the remains of children (0-3 years) radiocarbon dated to the 17th-19th c. (unpublished report). These were not included in the study because of their recent chronology into the early modern period and young age. Juveniles exhibit enriched $\delta^{13}C$ and $\delta^{15}N$ values relative to adults, due to trophic effects from gestation and nursing[54,55]. Children also tend to eat different foods than adults[56].

An individual recovered from an 8th-10th c. kurgan-style burial was included in this study. The low-lying stone mound was located seven meters from the cluster of early modern burials. A looted context was found that contained disarticulated remains of a female individual (KZ-BR5), which was directly dated to cal AD 773-916 (2-sigma; OS-72514; 1160 ± 30 BP), which is first reported here. The other two medieval individuals that were included in this study were excavated in 2006. Two individuals (Karatal BR-1 and BR-2) were excavated after discovering the remains were partially exposed and threatened by an advancing erosional cut in the topsoil and deeper strata. Karatal BR-2 was directly radiocarbon dated to cal AD 972-1032[57], and Karatal BR-1 is presumed to be from the same period based on stratigraphic positioning and associated horse riding tack and iron weapons, similar to those found in burials in the medieval period[51]. Around one of the leg bones of the Karatal BR-2 individual, the excavators found a cooper band that had preserved two small pieces of woven cotton[57].

The finding of cotton fabrics suggests that cotton may have been an exchange good along the Silk Roads during the 10th-11th c., as it is possible that cotton could not have been grown as far north as Zhetysu[57]. However, medieval agriculture in Zhetysu may have supported water demanding crops such as cotton, on account of rice being grown to this day in the Karatal River watershed[58] and rice grains were recovered from medieval sites in the Otrar Oasis[25]. The only subsistence data recovered from excavated Karatal burials included a small number of sheep bones in KZ-BR5 that represent one individual animal, and a horse burial next to Karatal BR-1 that was not fully excavated.

Turkmenistan

Dehistan Plain

Geoktchik Depe is an urban complex of approximately 5.5 hectares that comprises two main features: a large mound that contains much of the Iron Age occupation (13th-8th c. BC) and a rectangular structure that is associated with the Sassanid and Islamic period (6th-16th c.)[59]. The site contains a rich faunal assemblage, which has been published in detail, and suggests a strong pastoral component to subsistence[59]. Notably, a small number of fish and bird bones were recovered from several phases of the site[59], which had been incorrectly reported in a paleodietary study presenting a stable isotope analysis[60]. The site is associated with a complex network of irrigation canals that likely supported intensive farming[59], which appears to have been dominated by C_3 crops (wheat and barley) that primarily contributed to human diets at the site[60].

Misrijan is not well described in the English archaeological literature beyond a mention of being a 'medieval town'[59], and a search for specific information about the site was unsuccessful. Human and fauna carbon and nitrogen isotopic values are reported from the site, but cultural details are not provided[60]. There is a detailed monograph in Russian that describes the medieval archaeological sites on the Dehistan Plain[61]. However, the naming of the site under analysis as 'Misrijan' by Bocherens et al.[60] may be confused with the Misrijan Oasis, which contains dozens of large medieval settlements and precludes identifying the precise site at this time.

Supplementary Figure S1: Boxplots of carbon and nitrogen stable isotopic ratios from all regions and periods.

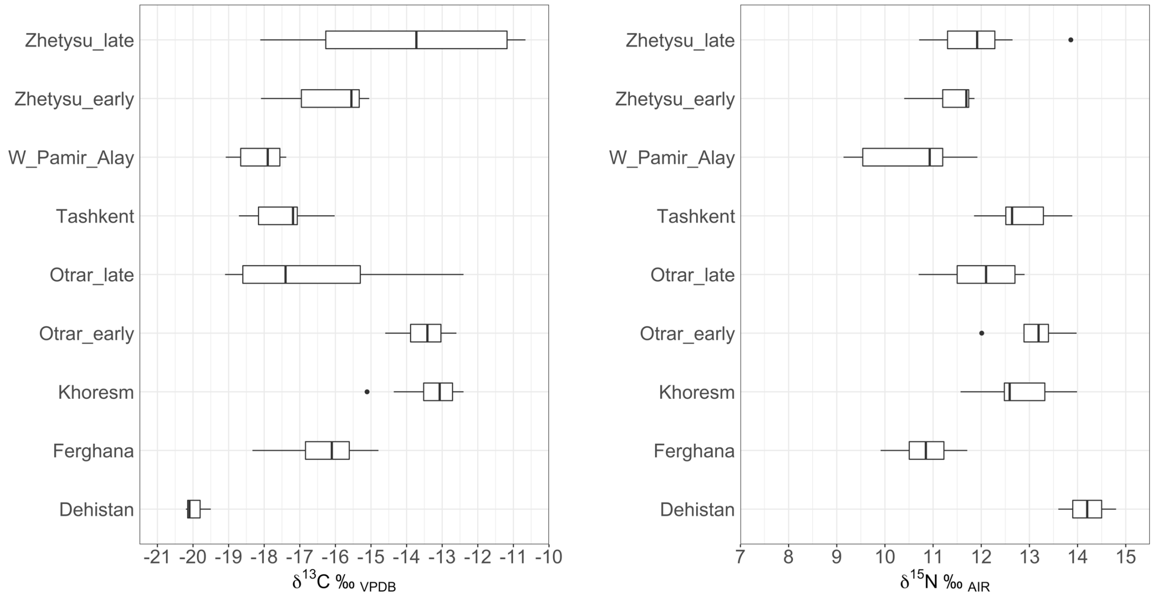

Supplementary Table S1: Summary statistics of human isotopic values

Country	Site/Region	n	Chronology	δ13C (‰) VPDB Min	Max	Median	Mean	Sd	δ15N (‰) AIR Min	Max	Median	Mean	Sd
Uzbekistan													
	**Zerafshan Basin**	**9**		**-19.08**	**-17.38**	**-17.90**	**-18.12**	**0.67**	**9.14**	**11.92**	**10.93**	**10.52**	**1.02**
	Tashbulak	4	10th-11th c.	-19.02	-17.38	-18.14	-18.17	0.80	11.17	11.92	11.31	11.43	0.35
	Altyntepe	1	10th-13th c.	-17.56	-	-	-	-	9.14	-	-	-	-
	Frinkent	4	10th-13th c.	-19.08	-17.54	-18.12	-18.21	0.66	9.33	10.93	9.81	9.97	0.71
	**Ferghana Valley**	**15**		**-18.33**	**-14.79**	**-16.10**	**-16.28**	**0.91**	**9.91**	**11.71**	**10.85**	**10.80**	**0.53**
	Chor Dona	4	11th-13th c.	-18.33	-16.68	-17.04	-17.27	0.73	9.91	11.71	10.47	10.64	0.82
	Chartok	11	12th c.	-17.17	-14.79	-15.69	-15.92	0.68	10.00	11.34	10.92	10.86	0.43
	**Tashkent Oasis**	**9**		**-15.11**	**-12.40**	**-13.07**	**-13.34**	**0.88**	**11.57**	**13.99**	**12.59**	**12.81**	**0.70**
	Uturlik	9	12th c.	-18.71	-16.02	-17.19	-17.48	0.85	11.85	13.89	12.64	12.78	0.67
	**Khoresm**	**9**		**-18.71**	**-16.02**	**-17.19**	**-17.48**	**0.85**	**11.85**	**13.89**	**12.64**	**12.78**	**0.67**
	Tok-Kala	9	8th-13th c.	-15.11	-12.40	-13.07	-13.34	0.88	11.57	13.99	12.59	12.81	0.70
Kazakhstan													
	**Otrar Oasis**	**13**		**-19.10**	**-12.40**	**-15.30**	**-15.72**	**2.51**	**10.70**	**13.98**	**12.10**	**12.32**	**0.92**
	Konyr-Tobe I	9	5th-7th c.	-19.10	-12.40	-17.40	-16.70	2.37	10.70	12.90	12.10	11.98	0.77
	Temirlanovka	4	2nd-4th c.	-14.59	-12.60	-13.42	-13.51	0.84	12.01	13.98	13.19	13.09	0.81
	**Zhetysu**	**15**		**-18.11**	**-10.66**	**-15.46**	**-15.03**	**2.58**	**10.40**	**13.86**	**11.71**	**11.71**	**0.83**
	Turgen II	7	2nd-6th c.	-18.09	-15.05	-15.55	-16.18	1.17	10.40	11.86	11.69	11.40	0.53
	Butakty II	6	10th-12th c.	-15.70	-10.66	-11.65	-12.69	2.24	10.71	12.16	11.51	11.54	0.56
	Karatal	2	10th-11th c.	-18.11	-17.98	-	-	-	12.65	13.86	-	-	-
Turkmenistan													
	**Dehistan Plain**	**3**		**-20.10**	**-19.50**	**-**	**-19.90**	**0.35**	**13.60**	**14.80**	**-**	**14.20**	**0.60**
	Geotchik Depe	2	Iron Age	-20.10	-20.10	-	-	-	13.60	14.20	-	-	-
	Misrijan	1	11th-12th c.	-19.50	-	-	-	-	14.80	-	-	-	-

Supplementary Table S2: Raw isotope data and information on human samples from medieval sites in Uzbekistan and Kazakhstan that were newly analysed in this study.

Lab ID	Archival ID	Original ID	Region	Site	Chronology	Skeletal element	Portion	Side	Sex	Age (years)	$\delta^{13}C$ (‰)	$\delta^{15}N$ (‰)	C:N	%C	%N
4945	Чартак7	3дв	Ferghana Valley	Chartok	12th c.	parietal	superior of eminence	left	Female	Middle/Old Adult (32-50+)	-15.54	11.34	3.19	47.04	17.21
4946	Чартак4	13дв	Ferghana Valley	Chartok	12th c.	parietal	superior of eminence	left	Female?	Young Adult (18-30)	-14.79	10.00	3.32	48.62	17.10
4947	Чартак19	23дв	Ferghana Valley	Chartok	12th c.	parietal	superior of eminence	right	Female	Middle Adult (30-45)	-15.69	10.92	3.25	46.95	16.84
4948	Чартак16	21дв	Ferghana Valley	Chartok	12th c.	mandible	medial surface	left	Male	Middle Adult (35-50)	-16.64	11.24	3.31	46.89	16.53
4949	Чартак9	15дв	Ferghana Valley	Chartok	12th c.	mandible	inferior of body	left	Male	Young/Middle Adult (23-44)	-15.81	11.21	3.27	47.32	16.88
4950	Чартак18	19дв	Ferghana Valley	Chartok	12th c.	parietal	superior of eminence	right	Female	Middle/Old Adult (30-50+)	-16.71	10.46	3.28	47.70	16.95
4951	Чартак17	18?	Ferghana Valley	Chartok	12th c.	parietal	superior of eminence	left	Female	Old Adult (50+)	-15.68	11.12	3.27	46.28	16.51
4952	Чартак3	16?	Ferghana Valley	Chartok	12th c.	parietal	superior of eminence	left	Unknown	Old Adult (50+)	-16.10	10.77	3.24	46.95	16.89
4953	Чартак1	12дв	Ferghana Valley	Chartok	12th c.	parietal	superior of eminence	left	Female	Middle/Old Adult (28-50+)	-15.53	11.27	3.25	46.66	16.75
4954	Чартак14	20дв	Ferghana Valley	Chartok	12th c.	parietal	superior of eminence	left	Male?	Young/Middle Adult (18-44)	-15.52	10.59	3.26	46.95	16.79
4955	Чартак11	11дв	Ferghana Valley	Chartok	12th c.	parietal	superior of eminence	left	Unknown	Old Adult (50+)	-17.17	10.55	3.30	46.60	16.47
4956	ЧД4	14-01, п.19	Ferghana Valley	Chor Dona	11th-13th c.	mandible	medial surface	left	Male	Young/Middle Adult (23-50)	-16.97	10.85	3.26	46.79	16.76
4958	ЧД5	14-05, п.14	Ferghana Valley	Chor Dona	11th-13th c.	mandible	medial surface	right	Male	Middle Adult (30-50)	-17.10	9.91	3.33	46.56	16.33
4959	ЧД3	14-06, п.17	Ferghana Valley	Chor Dona	11th-13th c.	mandible	medial surface	right	Male	Adult	-18.33	10.09	3.22	45.20	16.39
4960	ЧД1	14-06	Ferghana Valley	Chor Dona	11th-13th c.	temporal	zygomatic arch	right	Male	Adult	-16.68	11.71	3.15	44.35	16.44
4961	TBK J6 BR2 Indiv. 1	TBK J6 BR2 Indiv. 1	West Pamir-Alay	Tashbulak	10th-11th c.	tibia	medial proximal shaft	left	Female	Young/Middle Adult (27-50)	-19.08	10.93	3.15	46.43	17.18
4962	TBK F6 BR4 Indiv. 1	TBK F6 BR4 Indiv. 1	West Pamir-Alay	Tashbulak	10th-11th c.	tibia	medial proximal shaft	right	Female	Middle/Old Adult (30-50+)	-18.34	10.07	3.14	44.34	16.47
4963	TBK F6 BR5 Indiv. 1	TBK F6 BR5 Indiv. 1	West Pamir-Alay	Tashbulak	10th-11th c.	tibia	medial proximal shaft	right	Female	Young/Middle Adult (27-50)	-17.54	9.33	3.10	45.70	17.21
4964	TBK J6 BR1 Indiv. 1	TBK J6 BR1 Indiv. 1	West Pamir-Alay	Tashbulak	10th-11th c.	tibia	medial proximal shaft	right	Female	Old Adult (50+)	-17.90	9.54	3.18	46.13	16.91
4965	Фринкент4	N1	West Pamir-Alay	Frinkent	10th-13th c.	parietal	superior of eminence	right	Male	Middle/Old Adult (30-50+)	-19.02	11.92	3.18	43.30	15.87
4966	Фринкент5	N2	West Pamir-Alay	Frinkent	10th-13th c.	parietal	superior of eminence	left	Female	Old Adult (50+)	-17.61	11.20	3.19	45.69	16.69
4967	Фринкент3	N5	West Pamir-Alay	Frinkent	10th-13th c.	parietal	superior of eminence	left	Female	Middle/Old Adult (30-50+)	-18.66	11.43	3.23	45.88	16.56
4968	Фринкент1	N3	West Pamir-Alay	Frinkent	10th-13th c.	parietal	superior of eminence	right	Male	Middle/Old Adult (30-50+)	-17.38	11.17	3.31	48.45	17.09
4970	Алтун1	03-85, N2	West Pamir-Alay	Altyntepe	10th-13th c.	parietal	superior of eminence	right	Male?	Middle Adult (30-50)	-17.56	9.14	3.32	46.54	16.34

Supplementary Table S2: Raw isotope data and information on human samples from medieval sites in Uzbekistan and Kazakhstan that were newly analysed in this study.

4971	УТ8	п.37	Tashkent Oasis	Uturlik Tepe	10th-12th c.	parietal	superior of eminence	right	Unknown	Unknown	-16.82	13.29	3.39	47.05	16.17
4972	УТ1	п.22	Tashkent Oasis	Uturlik Tepe	10th-12th c.	mandible	medial surface	right	Male	Old Adult (50+)	-17.11	12.56	3.30	46.49	16.43
4973	УТ3	п.45	Tashkent Oasis	Uturlik Tepe	10th-12th c.	mandible	medial surface	right	Female?	Adult	-18.16	11.85	3.31	46.50	16.40
4974	УТ2	71, холма	Tashkent Oasis	Uturlik Tepe	10th-12th c.	temporal	zygomatic arch	right	Male?	Young/Middle Adult (18-44)	-18.22	12.51	3.27	47.50	16.96
4975	УТ7	п.39	Tashkent Oasis	Uturlik Tepe	10th-12th c.	mandible	inferior of body	right	Unknown	Unknown	-16.02	13.89	3.35	48.80	16.99
4976	УТ5	п.35	Tashkent Oasis	Uturlik Tepe	10th-12th c.	mandible	medial surface	right	Male	Young Adult (18-30)	-17.19	13.50	3.35	47.72	16.64
4977	УТ6	п.15	Tashkent Oasis	Uturlik Tepe	10th-12th c.	parietal	superior of eminence	right	Unknown	Unknown	-17.07	12.05	3.34	47.44	16.57
4978	УТ4	п.46	Tashkent Oasis	Uturlik Tepe	10th-12th c.	parietal	superior of eminence	right	Male	Middle/Old Adult (30-50+)	-18.03	12.70	3.32	46.81	16.47
4979	УТ9	п.46	Tashkent Oasis	Uturlik Tepe	10th-12th c.	mandible	inferior of body	right	Unknown	Unknown	-18.71	12.64	3.17	44.91	16.51
4980	TK12	п.15,N11	Khoresm	Tok Kala	9th-11th c.	parietal	superior of eminence	left	Female	Middle Adult (30-50)	-13.07	13.32	3.32	48.46	17.02
4982	TK15	п.40,N26	Khoresm	Tok Kala	9th-11th c.	parietal	superior of eminence	left	Male	Young/Middle Adult (18-44)	-12.71	11.57	3.22	46.82	16.95
4983	TK18	п.5,N25	Khoresm	Tok Kala	9th-11th c.	parietal	superior of eminence	left	Male?	Middle Adult (30-50)	-12.61	12.48	3.28	46.83	16.64
4984	TK5	п.20,N14	Khoresm	Tok Kala	9th-11th c.	parietal	superior of eminence	right	Male?	Middle Adult (30-50)	-15.11	13.99	3.33	48.33	16.92
4985	TK9	п.32,N31	Khoresm	Tok Kala	9th-11th c.	parietal	superior of eminence	left	Female?	Middle Adult (30-50)	-14.36	13.38	3.30	47.20	16.67
4986	TK23	п.15,N8	Khoresm	Tok Kala	9th-11th c.	parietal	superior of eminence	left	Male	Middle/Old Adult (30-50+)	-13.00	13.01	3.31	48.61	17.15
4987	Tk20	п.22,N3	Khoresm	Tok Kala	9th-11th c.	parietal	superior of eminence	left	Female?	Young Adult (20-30)	-13.30	12.53	3.29	47.78	16.93
4988	TK13	п.29,N9	Khoresm	Tok Kala	9th-11th c.	parietal	superior of eminence	left	Male?	Middle Adult (30-50)	-12.40	12.40	3.29	48.24	17.09
4989	TK1	п.8	Khoresm	Tok Kala	9th-11th c.	parietal	superior of eminence	left	Male	Old Adult (50+)	-13.52	12.59	3.30	45.94	16.26
6965	Karatal BR-2	Karatal BR-2	Zhetysu	Karatal	10th-11th c.	mandible	inferior of body	right	Unknown	Adult	-18.11	12.65	2.94	43.49	17.27
6966	Karatal BR-1	Karatal BR-1	Zhetysu	Karatal	10th-11th c.	tibia	medial proximal shaft	left	Male	Middle Adult (35-49)	-17.98	13.86	3.13	45.93	17.15
5164	KZ-BR5	KZ-BR5	Zhetysu	Karatal	8th-10th c.	femur	medial proximal shaft	right	Female	Middle Adult (35-49)	-16.20	12.39	3.10	45.49	17.07

Carbon isotope reference:
VPDB
Nitrogen isotope reference:
AIR

Supplementary Table S3: Raw isotope data of medieval human from south-eastern Kazakhstan previously published in Motuzaite Matuzeviciute et al.[43]

Lab ID	Original ID	Region	Site	Chronology	$\delta^{13}C$ (‰)	$\delta^{15}N$ (‰)	C:N
GM_139	Kurgan 1/grave 17	Otrar Oasis	Konyr-Tobe I	5th-7th c.	-15.3	12.7	3.33
GM_141	Kurgan 1/grave 15	Otrar Oasis	Konyr-Tobe I	5th-7th c.	-13.8	11.5	3.07
GM_142	Kurgan 1/grave16	Otrar Oasis	Konyr-Tobe I	5th-7th c.	-12.4	12.9	3.32
GM_145		Otrar Oasis	Konyr-Tobe I	5th-7th c.	-19.1	12.1	3.08
GM_146	Kurgan 1/grave 13	Otrar Oasis	Konyr-Tobe I	5th-7th c.	-18.2	10.7	3.11
GM_144	Kurgan 1/grave 6	Otrar Oasis	Konyr-Tobe I	5th-7th c.	-17.4	11.1	3.32
GM_147	Kurgan 1/grave 17	Otrar Oasis	Konyr-Tobe I	5th-7th c.	-16.8	12.1	3.09
GM_143		Otrar Oasis	Konyr-Tobe I	5th-7th c.	-18.7	12.8	3.09
GM_140	Kurgan 1/grave 14	Otrar Oasis	Konyr-Tobe I	5th-7th c.	-18.6	11.9	3.11
GM_001	Kurgan 20	Otrar Oasis	Temirlanovka	2nd-4th c.	-13.65	13.20	3.26
GM_002	Kurgan 26	Otrar Oasis	Temirlanovka	2nd-4th c.	-12.60	12.01	3.23
GM_005	Kurgan 33/grave 1	Otrar Oasis	Temirlanovka	2nd-4th c.	-13.18	13.18	3.19
GM_004	Kurgan 33/grave 2	Otrar Oasis	Temirlanovka	2nd-4th c.	-14.59	13.98	3.18
GM_007	Kurgan 10/grave 1	Zhetysu	Turgen II	2nd-6th c.	-16.65	11.72	3.41
GM_013	Kurgan 7/grave 1	Zhetysu	Turgen II	2nd-6th c.	-17.26	10.97	3.15
GM_009	Kurgan 10/grave 3	Zhetysu	Turgen II	2nd-6th c.	-15.05	11.86	3.52
GM_010		Zhetysu	Turgen II	2nd-6th c.	-18.09	10.40	3.10
GM_008	Kurgan 10/grave 2	Zhetysu	Turgen II	2nd-6th c.	-15.46	11.69	3.44
GM_012	Kurgan 8	Zhetysu	Turgen II	2nd-6th c.	-15.20	11.43	3.10
GM_006	Kurgan 9	Zhetysu	Turgen II	2nd-6th c.	-15.55	11.76	3.52
GM_ 015	Grave 16	Zhetysu	Butakty I	10th-12th c.	-10.66	11.32	3.15
GM_016	Kurgan 35/2	Zhetysu	Butakty I	10th-12th c.	-11.18	12.12	3.40
GM_018	Grave 23	Zhetysu	Butakty I	10th-12th c.	-12.11	11.71	3.22
GM_017	Grave 35/1	Zhetysu	Butakty I	10th-12th c.	-11.17	12.16	3.13
GM_014	Kurgan 1/grave 1	Zhetysu	Butakty I	10th-12th c.	-15.34	10.71	3.22
GM_003		Zhetysu	Butakty I	10th-12th c.	-15.70	11.24	3.14

Notes:

Isotope values are rounded to the nearest hundredth, while data from Konyr-Tobe I are reported to the nearest tenth, as previously published.

Skeletal element data were not provided in original source.

Archaeological contextual information is transcribed as published and placed in the "Original ID" column here.

Original Western Arabic numerals after site names have been replaced here with Roman numerals.

%C and %N were not reported.

Supplementary Table S4: Raw isotope data of medieval and Iron Age human isotopic data from Turkmenistan previously published in Bocherens et al.[60]

Sample ID	Region	Site	Chronology	Skeletal element	Age	$\delta^{13}C$ (‰)	$\delta^{15}N$ (‰)	C:N	%C	%N
MS 101	Dehistan	Misrijan	11th-12th c.	mandible	6/7 years	-19.5	14.8	3.2	45.0	16.5
GD 94/303	Dehistan	Geotchik Depe	Iron Age	mandible	adult	-20.1	14.2	3.2	25.9	9.3
GD 95/433	Dehistan	Geotchik Depe	Iron Age	talus	adult	-20.1	13.6	3.2	39.4	14.3

Supplementary Table S5: Rejected human samples from medieval sites in Uzbekistan

Lab ID	Archival ID	Original ID	Region	Site	Chronology	Skeletal element	Portion	Sex	Age (years)	Side	$\delta^{13}C$ (‰)	$\delta^{15}N$ (‰)	C:N	%C	%N	Comment
4957	ЧД2	14-07, п.16	Ferghana Valley	Chor Dona	11th-13th c.	mandible	medial surface	Male?	Young/Middle Adult (20-43)	right	-21.43	9.84	5.13	52.64	11.97	Excess carbon
4969	Фринкент2	п.4	West Pamir-Alay	Frinkent	10th-13th c.	frontal	-	Female?	Young Adult (20-30)	right	-	-	-	-	-	Failed extraction
4981	ТК19	п.37,N17	Khoresm	Tok Kala	9th-11th c.	parietal	superior of eminence	Female?	Young/Middle Adult (18-50)	left	-	-	-	-	-	Failed extraction

Carbon isotope reference: VPDB

Nitrogen isotope reference: AIR

Supplementary Information 4: $\delta^{13}C$ and $\delta^{15}N$ means and 95% CIs obtained from Bayesian bootstrapping ($mean_b$)

$\delta^{13}C$ $mean_b$

Region	Mean	2.50%	97.50%
W_Pamir_Alay	-18.12	-18.53	-17.76
Ferghana	-16.28	-16.74	-15.88
Tashkent	-17.49	-17.97	-16.98
Khoresm	-13.34	-13.91	-12.90
Otrar_late	-16.71	-17.94	-15.15
Otrar_early	-13.51	-14.18	-12.92
Zhetysu_early	-16.18	-16.98	-15.53
Zhetysu_late	-14.27	-16.00	-12.56
Dehistan	-19.93	-20.16	-19.61

$\delta^{15}N$ $mean_b$

Region	Mean	2.50%	97.50%
W_Pamir_Alay	10.53	9.94	11.10
Ferghana	10.80	10.53	11.05
Tashkent	12.78	12.40	13.18
Khoresm	12.81	12.39	13.23
Otrar_late	11.98	11.50	12.41
Otrar_early	13.09	12.44	13.66
Zhetysu_early	11.40	11.01	11.67
Zhetysu_late	12.01	11.50	12.60
Dehisan	14.20	13.73	14.67

Supplementary Information 5: Environmental modelling

Environmental conditions at sites were estimated from abiotic variables using an approach combining geographic information system (GIS) and redundancy analysis (RDA). Both 10 and 50 km analytical buffers were created around each site, which was used to calculate two sets of spatial means of various climatic and soil parameters derived from geographic data layers (Supplemental Tables S5-S6). Supplemental Fig. S2 shows examples of annual mean temperature, precipitation of wettest and driest month, and weight % of clay in soil. Climatic data were obtained from the WorldClim project (http://worldclim.org/), which describe biologically meaningful variations of temperature and precipitation in ca. 1 km resolution (bioclim)[62]. These data represent monthly climate records from 1970-2000. This period is similar in average temperature and precipitation to the 'Medieval Warm Period' during the 8th-13th c. in Central Asia[63–65]. However, the early medieval period (2nd-6th c.), which is chronologically represented in our dataset, coincides with the 'Dark Ages Cold Period' and is less comparable to modern environmental patterns[66]. Soil data were obtained from the Soil Grid project (http://isric.org/) to provide additional variables relevant for agricultural production[67]. The size of the buffers serves three purposes: 1) represent two spatial scales of human life experiences, either through direct movement and interaction or through contact with others living in this zone; 2) capture environmental variation in site environs caused by landscape fluctuations, such as mountains acting as rain shields and having steep, rocky terrain that changes over short distances, which was especially evident for areas near Tashbulak, Butakty, and Turgen; 3) overcome differences in spatial scales of the underlying geographic data.

Environmental parameters for each site buffer where associated with that site's $\delta^{13}C$ and $\delta^{15}N$ mean, min, and max values. The mean isotope values, as point estimates, were insufficient to represent sites expressing wide isotopic variation, such as Konyr-Tobe in $\delta^{13}C$ or Temirlanovka in $\delta^{15}N$; min and max values were included to consider values at the ends of wide isotopic distributions, which were used in RDA with the R package vegan[68]. In order to reduce the susceptibility of the RDA to false positives, statistical significance was set at $p < 0.01$. It is important to note that this figure is considered liberal by scientists from multiple fields, who recommend $p < 0.005$[69]. Environmental parameters were examined for correlation and plotted as a distance matrix using hierarchical clustering (Supplemental Fig. S3). Highly correlated parameters were removed, and the remaining parameters were accepted to be used in RDA:

10km	50km
elevation	elevation
bio2	bio1
bio3	bio2
bio4	bio3
bio6	bio4
bio9	bio15
bio12	CECSOL
CLYPPT	CLYPPT

Using these parameters, stable isotopic data were fitted to each RDA using the envfit() function in vegan. No statistical significant relationships were found at $p < 0.01$ (Supplemental Fig. S4). However, for the 10km dataset at $p < 0.05$ a negative correlation was found between mean $\delta^{13}C$ values and bio3 (isothermality), in addition to a positive

correlation was found with bio4 (temperature seasonality). For the 50km dataset, a negative correlation was also found between accepted environmental parameters and bio3.

To further explore possible relationships, multiple linear regressions were performed using stable isotopic data and accepted environmental parameters. Critically, the Bonferroni correction was applied to the level of significance in order to counteract the problem of conducting multiple, simultaneous statistical tests, which would otherwise increase the probability of finding an erroneous result[70]. An initial alpha value of 0.01 was divided by 8, representing the number of accepted environmental parameters, to get a new level of statistical significance of 0.00125. At this level, no linear relationships were identified. However, for accepted environmental datasets of both 10km and 50km, linear relationships were identified at uncorrected levels of significance. The strongest of these linear relationships were between mean $\delta^{13}C$ values and 10 km bio3 (slope = -0.57; R^2 = 0.483; p = 0.006) and mean $\delta^{15}N$ values and 10km elevation (slope = -0.002; R^2 = 0.325; p = 0.033), which are displayed in Supplemental Fig. S5.

Isothermality is a measure of the difference between day-to-night temperatures relative to the annual summer-to-winter temperatures. A value of 100 indicates that the diurnal temperature range is equal to the annual temperature range, and smaller values indicate lower temperature diurnal ranges against the annual range. C_4 plants, including millets used in agriculture, are well adapted to hot and arid climate[71]. Locations with lower isothermality values have higher human mean $\delta^{13}C$ values, but it is not clear why isothermality would correlate with human $\delta^{13}C$ values. The chances of this correlation being spurious is supported by a clustering of sites in the bi-plot in three groups along the trend line and an overall low range of isothermality values between ca. 27 and 35% (Supplemental Fig. S5), which suggests there is unlikely to be a dependent relationship with factors that would influence dietary intake of C_4 plants.

There also appears to be a weak, negative linear correlation between elevation and mean $\delta^{15}N$ values, but with a p-value of 0.033 and ca. 32.5% data explanation (R^2 = 0.325), confidence in this result should be checked. In hot and arid ecosystems, typical of lowland regions in Central Asia, plant $\delta^{15}N$ values increase due to nitrogen loss from soils[72]. Agricultural products in lowland zones, especially those downstream from further farming run-off, can also be enriched in ^{15}N from exogenous inputs such as fertilizers and animal waste[73–77]. Thus, it is not possible to separate these processes given the prevalence of highly modified agricultural landscapes in the medieval period of Central Asia. Furthermore, these results demonstrate that patterns of agricultural production and dietary intake are more likely to be minimally affected by inter-regional differences in environment that were modelled here.

Supplementary Table S6: 50 km environmental data used in regression and redundancy analysis that were derived from bioclimatic variables[62] and soil properties[67].

site	elevation	bio1	bio2	bio3	bio4	bio5	bio6	bio7	bio8	bio9	bio10	bio11	bio12	bio13	bio14	bio15	bio16	bio17	bio18	bio19	CECSOL	PHIHOX	ORCDRC	CLYPPT	SNDPPT
Altyntepe	496.7	15.3	14.7	35.4	971.7	35.8	-5.8	41.6	7.7	25.7	27.6	3.4	381.6	81.2	0.2	84.0	195.1	4.7	6.1	161.9	22.4	78.0	4.7	28.3	29.0
Butakty-2	1355.7	6.5	11.0	27.6	1078.1	25.7	-14.5	40.2	11.6	5.5	19.1	-7.3	427.0	68.7	16.3	47.8	177.6	56.7	114.7	61.0	24.8	70.9	17.5	27.9	28.4
Chartok	574.5	13.3	12.8	30.5	1023.4	32.7	-9.1	41.8	11.3	23.8	25.5	0.2	249.7	33.3	4.3	48.3	97.0	18.2	27.3	77.0	19.6	79.4	6.7	27.3	29.6
Chor Dona	564.0	13.5	12.7	29.9	1034.6	33.0	-9.4	42.3	8.4	24.1	25.8	0.1	269.5	35.5	5.1	47.7	101.9	19.5	27.0	87.7	18.9	77.8	9.3	29.3	27.2
Frinkent	580.7	13.9	14.1	34.3	973.6	33.7	-7.4	41.1	7.8	24.2	26.1	1.9	346.3	65.3	1.1	74.7	162.6	8.2	11.2	138.9	22.2	77.5	6.5	28.7	29.0
Geoktchik Depe	130.1	17.1	13.3	34.0	962.5	35.9	-3.1	39.0	12.2	28.8	28.8	5.3	194.2	32.3	4.1	54.7	78.4	15.1	15.1	65.6	17.2	81.1	2.5	22.2	39.6
Karatal	582.5	7.9	14.2	29.9	1222.7	30.6	-16.9	47.5	8.2	21.2	22.4	-7.7	332.9	38.5	14.0	28.9	105.8	56.3	69.4	73.2	28.1	78.0	9.5	28.8	27.7
Konyr-Tobe-1	182.7	12.8	14.4	30.9	1168.2	35.0	-11.6	46.6	5.1	25.2	27.2	-1.8	221.8	33.7	1.0	61.9	87.9	9.0	12.0	86.3	27.6	79.6	8.1	30.4	31.9
Misrijan	189.5	16.5	12.9	33.8	946.7	34.9	-3.1	38.0	13.4	28.1	28.1	4.9	195.8	32.0	4.4	54.3	79.8	16.1	16.1	65.3	17.6	80.9	3.1	24.7	35.5
Tashbulak	1872.2	8.5	12.3	33.6	879.4	26.7	-9.8	36.5	7.6	17.7	19.6	-2.1	406.2	74.0	7.5	61.2	194.2	35.4	51.4	99.4	25.2	71.0	15.1	28.0	33.7
Temirlanovka	318.8	13.2	14.9	32.8	1105.7	34.8	-10.6	45.4	6.9	25.1	26.8	-0.8	340.3	51.3	2.2	61.2	135.1	12.5	17.4	122.2	27.2	76.6	6.3	33.3	27.2
Tok Kala	62.5	11.8	12.4	27.7	1195.4	33.2	-11.6	44.8	12.7	24.0	26.3	-3.0	105.4	16.3	2.0	50.8	45.0	8.0	10.3	31.3	22.6	81.3	7.1	29.9	27.7
Turgen-2	1276.8	6.8	11.9	30.2	1029.0	26.1	-13.4	39.5	13.7	-5.4	19.0	-6.0	469.0	67.0	19.3	39.6	180.8	65.5	135.9	66.0	24.8	69.4	17.1	26.1	29.1
Uturlik	271.0	14.7	14.2	33.6	1006.3	34.9	-7.4	42.3	9.1	27.0	27.0	2.0	316.0	50.1	1.0	69.3	140.6	7.6	7.6	124.2	20.5	78.2	9.0	30.1	27.1

Supplemental Table S7: 10 km environmental data used in regression and redundancy analysis that were derived from bioclimatic variables[62] and soil properties[67].

Site	elevation	bio1	bio2	bio3	bio4	bio5	bio6	bio7	bio8	bio9	bio10	bio11	bio12	bio13	bio14	bio15	bio16	bio17	bio18	bio19	CECSOL	PHIHOX	ORCDRD	CLYPPT	SNDPPT
Altyntepe	474.7	15.2	14.4	35.3	954.7	35.5	-5.2	40.7	7.4	25.4	27.3	3.6	380.8	81.2	0.0	84.0	194.3	4.6	6.0	162.3	20.1	77.8	6.0	27.9	29.5
Butakty-2	1154.0	7.7	10.7	26.4	1104.2	27.2	-13.4	40.6	14.2	1.8	20.5	-6.6	423.1	68.6	17.9	44.2	171.5	62.8	102.6	63.9	24.9	72.8	14.9	28.9	26.7
Chartok	544.7	13.5	12.7	30.3	1028.2	32.9	-9.1	42.0	11.5	23.9	25.7	0.2	240.5	32.0	3.9	48.2	92.3	16.7	25.6	75.5	18.4	79.3	6.5	26.6	30.7
Chor Dona	579.6	13.5	12.7	29.7	1038.2	33.3	-9.4	42.6	8.3	24.0	25.9	0.1	274.5	36.4	5.3	47.6	103.9	19.9	27.7	88.9	18.2	78.1	9.1	29.1	29.2
Frinkent	535.5	14.0	14.1	34.2	977.2	33.8	-7.4	41.2	8.2	24.3	26.2	1.9	341.4	64.9	1.0	75.5	161.1	7.5	10.2	138.0	21.0	78.1	6.4	28.5	28.3
Geoktchik Depe	127.1	17.2	13.3	33.8	966.1	36.2	-3.0	39.2	12.7	29.0	29.0	5.3	192.8	32.2	4.0	54.6	77.9	15.0	15.0	64.7	17.9	81.1	2.6	21.3	41.5
Karatal	621.2	8.0	14.0	30.4	1184.6	30.4	-15.8	46.2	7.6	21.1	22.1	-6.8	347.0	39.9	15.1	28.8	110.2	59.4	73.1	75.3	28.5	77.8	7.9	29.6	26.9
Konyr-Tobe-1	177.1	12.8	14.5	31.0	1168.1	35.0	-11.7	46.7	6.1	25.2	27.2	-1.8	222.5	33.5	1.0	61.5	87.3	9.0	12.0	86.4	27.9	79.8	9.0	30.7	31.2
Misrijan	168.3	16.3	12.7	33.6	945.1	34.7	-3.2	37.9	13.2	27.9	27.9	4.8	197.4	32.5	4.3	54.9	80.4	15.7	15.7	65.9	16.9	81.0	3.1	25.2	33.7
Tashbulak	1802.6	9.2	12.3	33.6	875.2	27.5	-9.0	36.5	8.6	19.2	20.2	-1.5	399.8	76.1	6.9	63.6	197.7	32.1	44.5	93.7	27.1	72.1	14.4	30.3	31.3
Temirlanovka	317.2	13.2	15.0	32.9	1105.8	35.0	-10.6	45.6	6.9	25.2	26.9	-0.7	340.9	51.6	2.0	61.2	135.3	12.2	17.3	122.1	27.2	76.5	5.4	33.5	26.6
Tok Kala	61.3	11.6	12.0	27.4	1182.4	32.7	-11.1	43.8	12.6	23.7	26.0	-2.9	103.8	15.8	2.0	50.3	44.1	8.0	10.3	31.1	23.8	81.5	6.5	30.0	27.5
Turgen-2	1041.4	8.2	11.7	29.3	1051.0	27.8	-12.1	39.8	14.4	-5.0	20.6	-5.0	456.5	65.9	20.2	37.1	173.1	67.8	118.5	67.8	24.7	70.6	17.7	28.0	26.1
Uturlik	270.0	14.7	14.3	33.9	1003.1	34.8	-7.3	42.1	9.1	27.0	27.0	2.0	315.3	49.8	1.0	69.4	140.5	7.5	7.5	123.9	20.3	78.6	10.7	30.5	26.4

Bioclimatic variables:

bio1	Annual mean temperature
bio2	Annual mean diurnal range
bio3	Isothermality
bio4	Temperature seasonality (CV)
bio5	Max temperature of warmest month
bio6	Min temperature of coldest month
bio7	Annual temperature range
bio8	Mean temperature of wettest quarter
bio9	Mean temperature of driest quarter
bio10	Mean temperature of warmest quarter
bio11	Mean temperature of coldest quarter
bio12	Annual precipitation
bio13	Precipitation of wettest month
bio14	Precipitation of driest month
bio15	Precipitation seasonality (CV)
bio16	Precipitation of wettest quarter
bio17	Precipitation of driest quarter
bio18	Precipitation of warmest quarter
bio18	Precipitation of warmest quarter
bio19	Precipitation of coldest quarter

Soil properties:

CECSOL	Cation exchange capacity
PHIHOX	pH index in water solution
ORCDRC	organic carbon content
CLYPPT	% wt clay particles (<0.0002 mm)
SNDPPT	% wt sand particles (0.05-2 mm

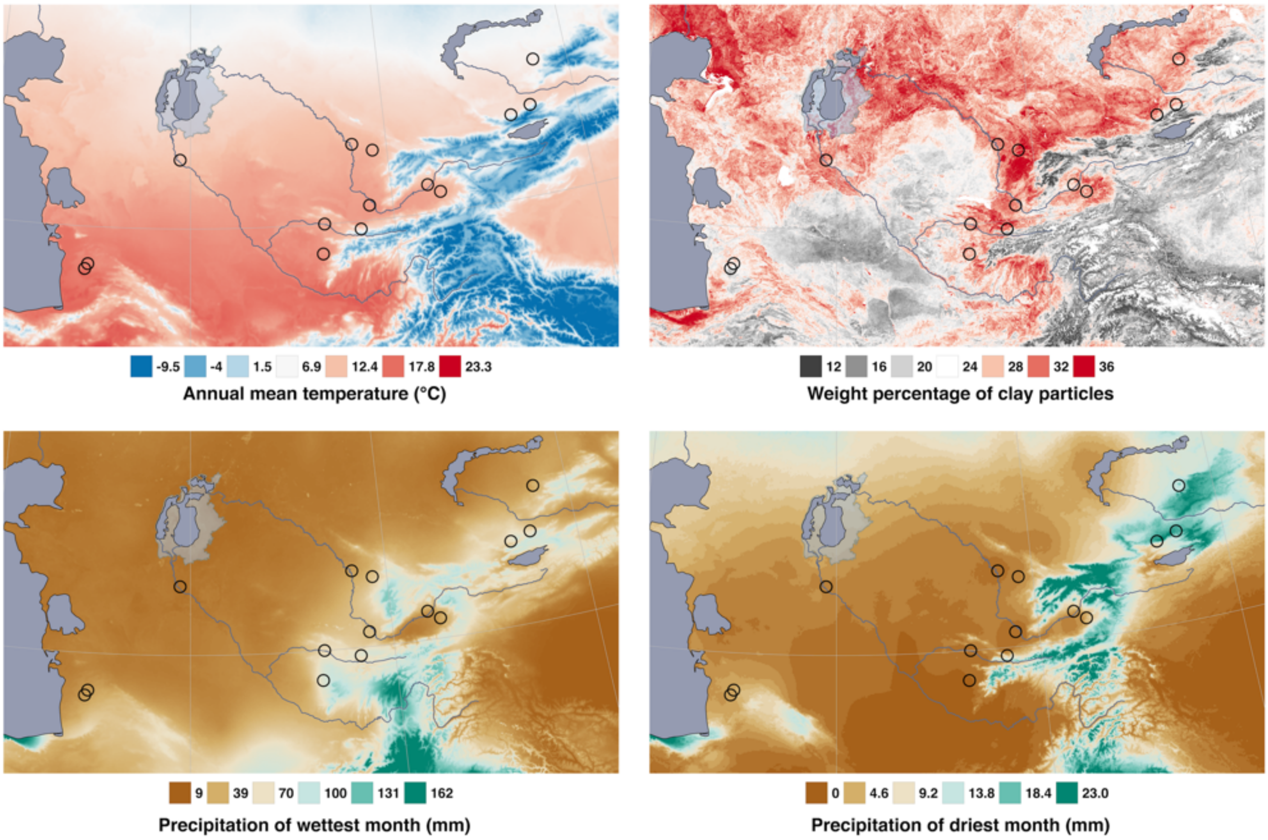

Supplementary Figure S2: Examples of geographic layers for environmental parameters and sites analysed in this study. Map scale and extent are identical to that in main text Fig. 1. Map generated with Quantum GIS, version 2.18.2 (https://www.qgis.org).

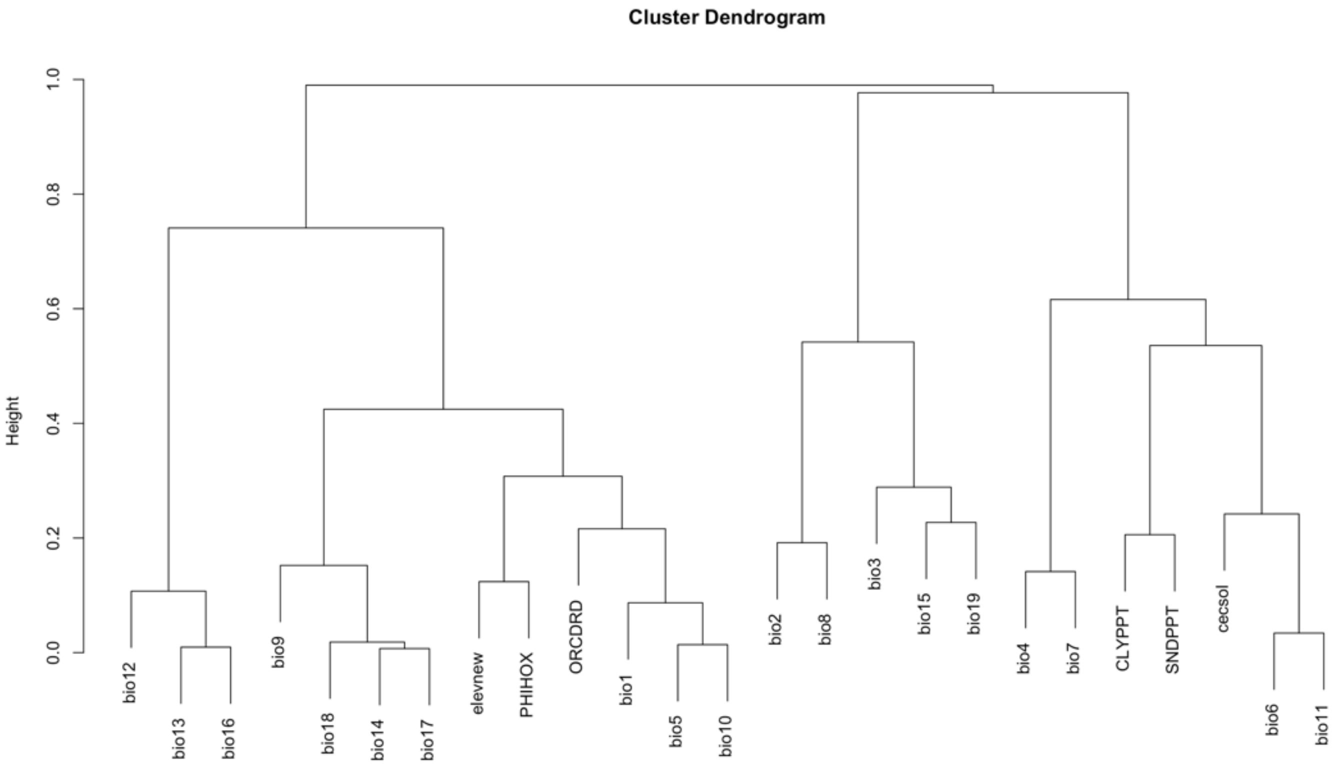

Supplementary Figure S3: Hierarchical clustering analysis of environmental variables derived from 10km buffers around sites.

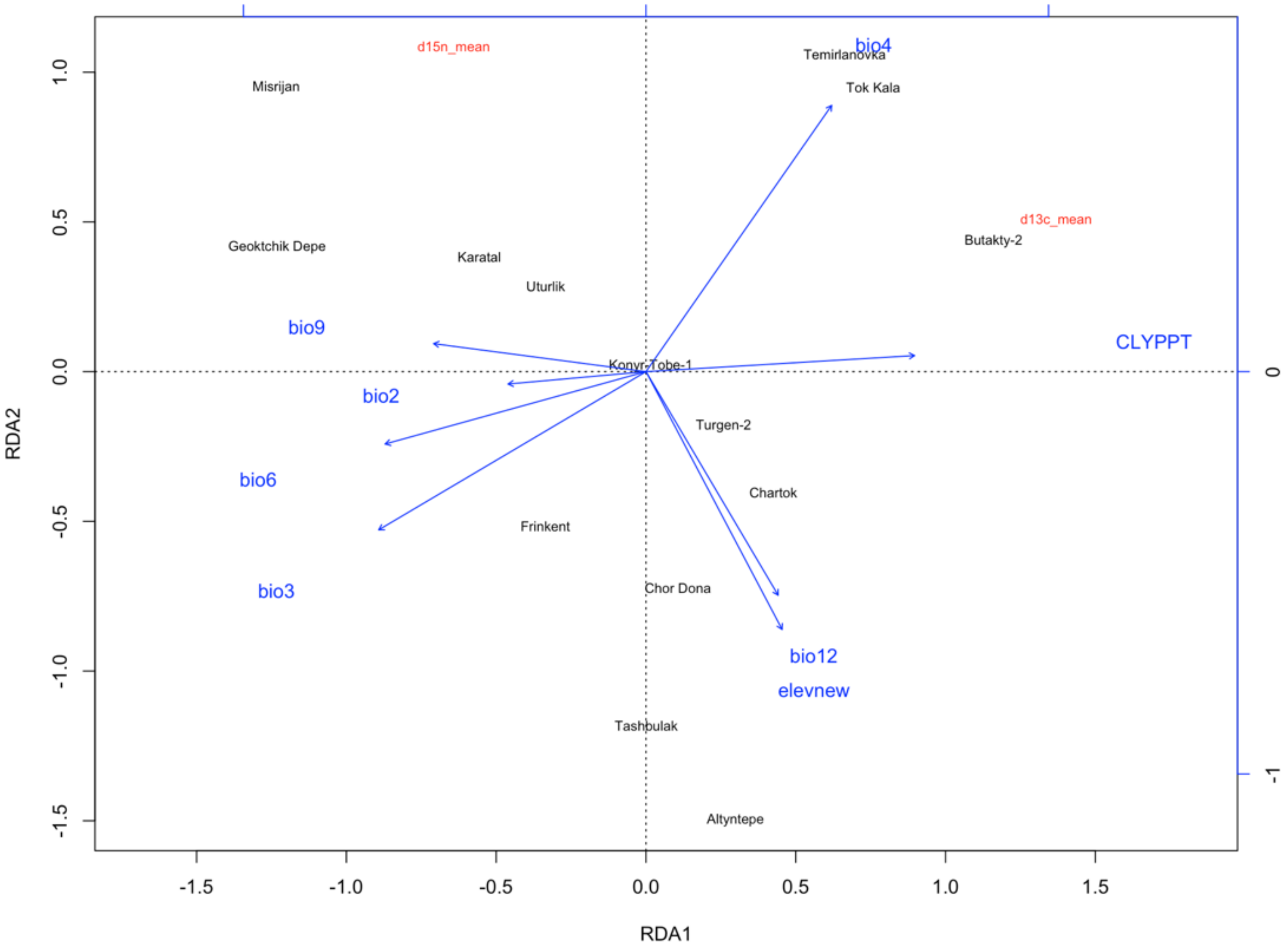

Supplementary Figure S4: RDA plot of stable isotope means per site and accepted site-level environmental parameters for 10km buffers.

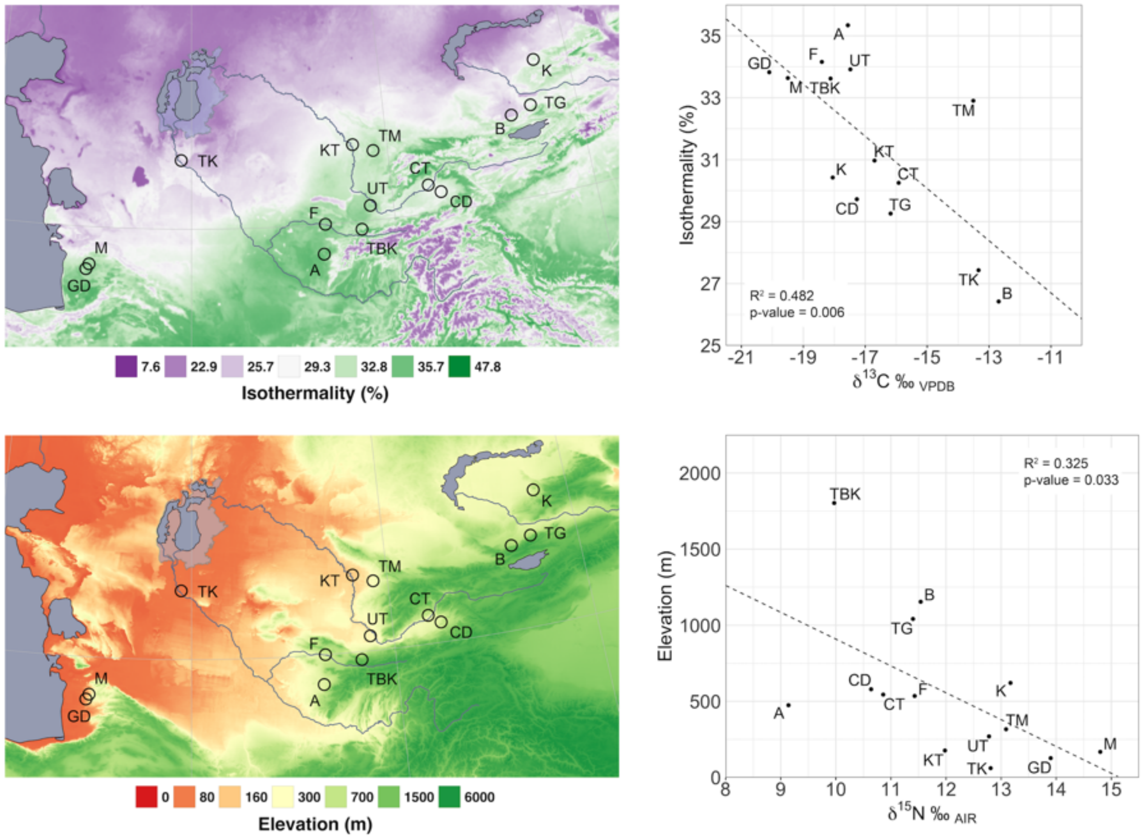

Supplementary Figure S5: Results of linear regression analysis between accepted environmental parameters and stable isotopic values. In the top panel, mean $\delta^{13}C$ values of each site moderately correlate with site isothermality (bio3). In the bottom panel, mean $\delta^{15}N$ values of each site weakly correlate with site elevation. The p-values here do not cross the level of significance after applying the Bonferroni correction. Site key: TK = Tok-kala, UT = Uturlik, CD = Chor Dona, CT = Chartok, TBK = Tashbulak, A = Altyntepe, F = Frinkent, GD = Geoktchik Depe, M = Misrijan, KT = Konyr-Tobe, TM = Temirlanovka, TG = Turgen, B = Butakty, K = Karatal. Map generated with Quantum GIS, version 2.18.2 (https://www.qgis.org).

Supplementary Information 6: Numerical output of isotopic niche modelling

SIBER Bayesian ellipse areas (‰^{2}) that are depicted in main text Fig. 3:

Otrar (early)
Mode 1.2 Mean 2.04 Median 1.66
95 % lower = 0.441 upper = 4.5
75 % lower = 0.678 upper = 2.51
50 % lower = 0.863 upper = 1.84

Otrar (late)
Mode 4.91 Mean 6.21 Median 5.73
95 % lower = 2.66 upper = 10.8
75 % lower = 3.39 upper = 7.67
50 % lower = 3.95 upper = 6.37

Zhetysu (early)
Mode 1.19 Mean 1.56 Median 1.4
95 % lower = 0.575 upper = 2.89
75 % lower = 0.754 upper = 1.94
50 % lower = 0.899 upper = 1.56

Zhetysu (late)
Mode 7.26 Mean 9.04 Median 8.35
95 % lower = 3.87 upper = 15.7
75 % lower = 4.95 upper = 11.2
50 % lower = 5.77 upper = 9.3

Dehistan
Mode 0.425 Mean 0.839 Median 0.629
95 % lower = 0.13 upper = 2.05
75 % lower = 0.211 upper = 1
50 % lower = 0.282 upper = 0.693

Ferghana
Mode 1.4 Mean 1.57 Median 1.5
95 % lower = 0.846 upper = 2.44
75 % lower = 1.03 upper = 1.9
50 % lower = 1.15 upper = 1.65

Khoresm
Mode 1.28 Mean 1.59 Median 1.46
95 % lower = 0.68 upper = 2.76
75 % lower = 0.869 upper = 1.96
50 % lower = 1.01 upper = 1.63

Tashkent
Mode 1.34 Mean 1.67 Median 1.54
95 % lower = 0.713 upper = 2.9
75 % lower = 0.911 upper = 2.06
50 % lower = 1.06 upper = 1.71

West Pamir-Alay
Mode 1.71 Mean 2.13 Median 1.96
95 % lower = 0.91 upper = 3.7
75 % lower = 1.16 upper = 2.63
50 % lower = 1.35 upper = 2.18

Supplementary Information 7: Isotopic niche overlap of urban medieval regions in Uzbekistan and Turkmenistan

SIBER Bayesian ellipse overlap analysis depicted in main text Figure 3, (Dehistan included below):

Regional pairs	Mean	Lower 95% CI	Upper 95% CI
West Pamir-Alay - Ferghana	18.8%	5.3%	35.5%
West Pamir-Alay - Tashkent	15.7%	3.2%	32.1%
West Pamir-Alay - Khoresm	0.0%	0.0%	0.0%
West Pamir-Alay - Dehistan	1.3%	0.0%	11.1%
Ferghana - Dehistan	0.0%	0.0%	0.0%
Ferghana - Khoresm	0.7%	0.0%	6.9%
Tashkent - Khoresm	2.2%	0.0%	12.9%
Tashkent - Ferghana	4.1%	0.0%	19.4%
Tashkent - Dehistan	1.1%	0.0%	11.7%
Khoresm - Dehistan	0.0%	0.0%	0.0%

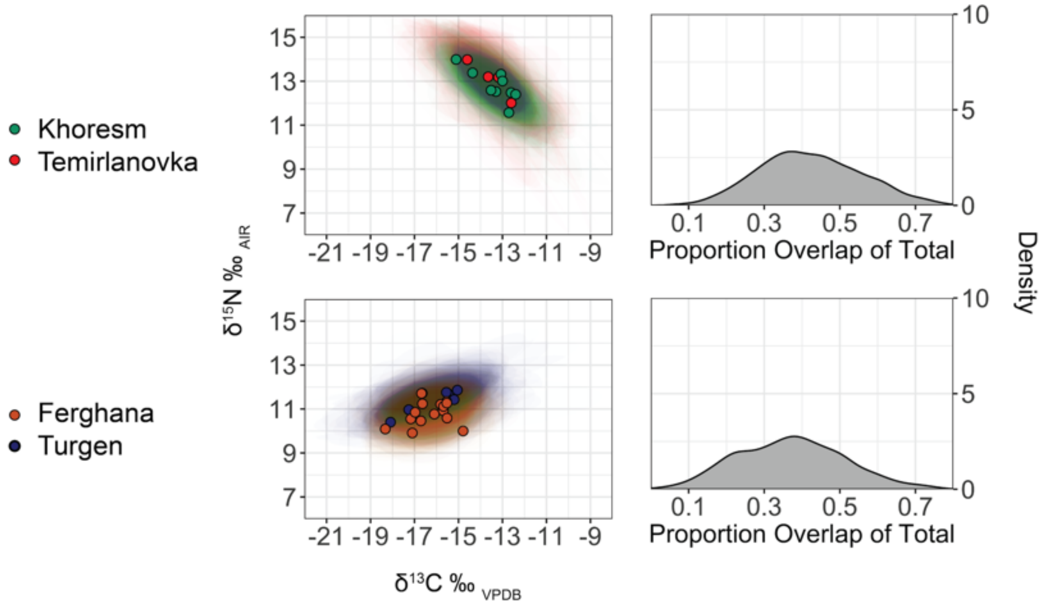

Supplementary Figure S6: Isotopic niche overlap between Khoresm and Temirlanovka (early Otrar) (top) and between Ferghana and Turgen (early Zhetysu) (bottom).

References

1. Barisitz, S. *Central Asia and the Silk Road: Economic Rise and Decline over Several Millennia*. (Springer, 2017).
2. Davidovich, E. A. The Karakhanids. in *History of civilizations of Central Asia, Volume 4 (Part 1)* (eds. Asimov, M. E. & Bosworth, C. E.) 125–149 (Unesco Publishing, 1998).
3. Rowton, M. B. Dimorphic Structure and the Parasocial Element. *J. East. Stud.* **36,** 181–198 (1977).
4. Hammer, E. L. & Arbuckle, B. 10,000 years of pastoralism in Anatolia: a review of evidence for variability in pastoral lifeways. *Nomadic Peoples* **21,** 214–267 (2017).
5. Hansen, V. *The Silk Road: a new history*. (Oxford University Press, 2012).
6. Biran, M. The Qarakhanids' Eastern Exchange: Preliminary Notes on the Silk Roads in the eleventh and twelfth Centuries. in *Complexity of Interaction along the Eurasian Steppe Zone in the First Millennium CE* (eds. Jan, B. & Michael, S.) **7,** 575–595 (Vor- und Frühgeschichtliche Archäologie Rheinische Friedrich-Wilhelms-Universität Bonn, 2015).
7. Golden, P. The Karakhanids and early Islam. in *The Cambridge History of Early Inner Asia* (ed. Sinor, D.) 343–370 (Cambridge University Press, 1990).
8. Negmatov. The Samanid State. in *History of civilizations of Central Asia, Volume 4 (Part 1)* (eds. Asimov, M. E. & Bosworth, C. E.) 77–94 (Unesco Publishing, 1998).
9. Maksudov, F. *et al.* Nomadic Urbanism at Tashbulak: A New Highland Town of the Qarakhanids. in *Central Asian Urbanism* (eds. Baumer, C. & Ecklin, S.) (in press).
10. Boroffka, N. G. O. *et al.* Human Settlements on the Northern Shores of Lake Aral and Water Level Changes. *Mitig. Adapt. Strateg. Glob. Change* **10,** 71–85 (2005).
11. Bilalov, A. I. *Iz istorii irrigatsii Ustrushany*. (Izd-vo 'Donish', 1980).
12. Groshev, V. A. *Irrigatsiya yuzhnogo Kazakhstana v sredniye veka*. (Izd-vo 'Nauka' KSSR, 1985).
13. Kasai, N. & Natsagdorj, S. Socio-economic Development: Food and Clothing in Eastern Iran and Central Asia. in *History of civilizations of Central Asia, Volume 4 (Part 1)* (eds. Asimov, M. E. & Bosworth, C. E.) 381–390 (Unesco Publishing, 1998).
14. Malatesta, L. C. *et al.* Dating the Irrigation System of the Samarkand Oasis: A Geoarchaeological Study. *Radiocarbon* **54,** 91–105 (2012).
15. Mukhamejanov, A. R. Socio-economic Development: Food and Clothing in Eastern Iran and Central Asia. in *History of civilizations of Central Asia, Volume 4 (Part 2)* (eds. Asimov, M. E. & Bosworth, C. E.) 275–297 (Unesco Publishing, 1998).
16. Saidov, A., Anarbaev, A. & Goriyacheva, V. The Ferghana Valley: The Pre-Colonial Legacy. in *Ferghana Valley: The Heart of Central Asia* (ed. Starr, S. F.) 3–28 (M.E. Sharpe, 2011).
17. Starr, S. F. *Lost enlightenment: Central Asia's golden age from the Arab conquest to Tamerlane*. (Princeton University Press, 2013).
18. Brykina, G. A. *Karabulak*. (Izd-vo 'Nauka', 1974).
19. Brykina, G. A. *Yugo-Zapadnaya Fergana v pervoj polovinye I tysyacheletiya nashej ery*. (Izd-vo 'Nauka', 1982).
20. Nerazik, E. E. *Sel'skiye poseleniya Afrigidskogo Khorezma*. (Izd-vo 'Nauka', 1966).
21. Gudovka, A. V. *Tok-kala*. (Izdatel'stvo Nauka Uzbekskoj SSR, 1964).
22. Tolstov, S. P. *Drevnij Khorezm*. (Izdanie MGU, 1948).
23. Anarbaev, A. Srednevekovoe zhilische Akhsikenta (XI-XII vv.). in *Istoriya Material'noj Kul'tury Uzbekistana* **35,** 220–229 (Izd-vo 'FAN', 2006).
24. Yakubov, Y. *Pargar v VII-VIII vekakh nashej ery*. (Izd-vo 'Donish', 1979).
25. Bashtannik, S. V. Archaeobotanical Studies at Medieval Sites in the Arys River Valley. *Archaeol. Ethnol. Anthropol. Eurasia* **33,** 85–92 (2008).
26. Brite, E. B. & Marston, J. M. Environmental change, agricultural innovation, and the spread of cotton agriculture in the Old World. *J. Anthropol. Archaeol.* **32,** 39–53 (2013).
27. Bubnova, M. A. K voprosu o zemledelii na zapadnom Pamire v IX-XI vv. in *Proshloe Srednej Azii: Arkheologiya, Numizmatika i Epigrafika, Etnografiya* 59–66 (Donish, 1987).

28. Gorbunova, N. G. *The Culture of Ancient Ferghana: VI Century B.C. - VI Century A.D.* (British Archaeological Reports, 1986).
29. Wu, X., Miller, N. F. & Crabtree, P. Agro-Pastoral Strategies and Food Production on the Achaemenid Frontier in Central Asia: A Case Study of Kyzyltepa in Southern Uzbekistan. *Iran* **53,** 93 (2015).
30. Brite, E. B., Khozhaniyazov, G., Marston, J. M., Cleary, M. N. & Kidd, F. J. Kara-tepe, Karakalpakstan: Agropastoralism in a Central Eurasian Oasis in the 4th/5th century A.D. Transition. *J. Field Archaeol.* **0,** 1–16 (2017).
31. Buryakov, Y. F. *Genezis i etapi razvitiya gorodskoj kul'tury Tashkentskogo oazisa*. (Izdatel'stvo Nauka Uzbekskoj SSR, 1982).
32. Brite, E. B. Irrigation in the Khorezm oasis, past and present: a political ecology perspective. *J. Polit. Ecol.* **23,** 2 (2016).
33. Frumkin, G. *Archaeology in Soviet Central Asia*. (Brill, 1970).
34. Khodzhajov, T. K. *Ethnicheskie Protsessy v Srednej Azii v Epokhu Srednevekov'ya*. (Izdatel'stvo Nauka Uzbekskoj SSR, 1987).
35. Buryakov, Y. F., Rostovtsev, O. M., Perevozchikov, I. V., Khodzhajov, T. K. & Khalilov, K. Mogil'nik Uturlik-Tepe. in *Uspekhi Sredneaziatskoj Arkheologii* (ed. Bochever, V. T.) **4,** 91 (Izd-vo 'Nauka', 1979).
36. Abdulgazieva, B. Issledovanie poseleniya Chordona. in *Istoriya material'noj kul'tury Uzbekistana* **25,** 132–137 (Izd-vo 'FAN', 1991).
37. Frachetti, M. D. & Maksudov, F. The landscape of ancient mobile pastoralism in the highlands of southeastern Uzbekistan, 2000 b.c.–a.d. 1400. *J. Field Archaeol.* **39,** 195–212 (2014).
38. Stark, S., Ėshonkulov, U., Gütte, M. & Rakhimov, N. Resource exploitation and settlement dynamics in high mountain areas: the case of medieval Ustrūshana (northern Tadzhikistan). *Archäol. Mitteilungen Aus Iran Turan* 67–85 (2010).
39. Lunina, S. B. *Goroda Yuzhnogo Sogda v VIII-XII vv.* (Izd-vo 'FAN', 1984).
40. Grigor'ev, G. V. Zoroastrijskoe kostekhranilische v kishlake Frinkent pod g. Samarkandom. *Vestn. Drevnej Istor.* **2,** 144–150 (1939).
41. Grenet, F. Zoroastrianism in Central Asia. in *The Wiley Blackwell Companion to Zoroastrianism* (eds. Stausberg, M. & Vevaina, Y. S.-D.) 129–146 (Wiley Blackwell, 2015).
42. Baipakov, K. M., Smagulov, E. A. & Erzhigitova, A. A. *Rannesrednevekovye Nekropoli Yuzhnogo Kazakhstana*. (BAUR, 2005).
43. Motuzaite Matuzeviciute, G. *et al.* The extent of cereal cultivation among the Bronze Age to Turkic period societies of Kazakhstan determined using stable isotope analysis of bone collagen. *J. Archaeol. Sci.* **59,** 23–34 (2015).
44. Smagulov, E. A. & Erzhigitova, A. A. K izucheniyu pogrebal'nykh sooruzhenij Otyrarskogo oazisa. in *Voprosy Arkheologii Kazakhstana* (ed. Bejsenov, A. Z.) 142–164 (Institut arkheologii im A.Kh. Margulana, 2011).
45. Frachetti, M. D., Benecke, N., Mar'yashev, A. N. & Doumani, P. N. Eurasian pastoralists and their shifting regional interactions at the steppe margin: settlement history at Mukri, Kazakhstan. *World Archaeol.* **42,** 622–646 (2010).
46. Baipakov, K. M. & Sejtkaliev, M. K. *Nauchnyj otchet no. AR-12/95 o vypolnennykh nauchno-issledovatel'skikh rabotakh na panyatnikakh arkheologii, raspolozhennykh na uchastke 'Obkhod c. Temirlanovki avtomagistrali Zapadnaya Evropa - Zapadnyk Kitaj'*. (Archaeological Expertise, LLC, 2012).
47. Sejtkaliev, M. K. Kinzhal redkogo tipa iz mogil'nika Temirlanovka-1. *Izv. NAN RK* **3,** 60–70 (2013).
48. Goryachev, A. A. Arkheologicheskij kompleks Turgen'. Evolyutsiya drevnikh kul'tur. in *Archeologiya Kazakhstana v Epochu Nezavisimosti: Itogi, Perspektivy* (ed. Bajtanaev, B. A.) **1,** 256–266 (Institut arkheologii im A.Kh. Margulana, 2011).
49. Goryachev, A. A., Caraev, V. V. & Egorova, T. A. K voprosu o khozyajstvenno-kul'turnom pazvitii drevnego naseleniya Almaty. *Tsentralno-Aziat. Iskusstv. Zhurnal* **3,** 18–30 (2016).
50. Goryachev, A. A. Arkheologicheskie pamyatniki kompleksa Butakty I na yugo-vostochnoj okraine goroda Almaty. *Izv. NAN RK* **1,** 45–59 (2006).
51. Goryachev, A. A. & Motov, Y. A. Rezul'taty issledovanij arkheologicheskogo kompleksa Butakty-I na yugo-vostochnoj okranie goroda Almaty v 2007 gody. *Izv. NAN RK* **1,** 67–82 (2008).

52. Baipakov, K. M. & Mar'yashev, A. N. *Byan-Zhurek Petroglifs*. (Credo, 2008).
53. Frachetti, M. D. *Pastoralist landscapes and social interaction in bronze age Eurasia*. (University of California Press, 2008).
54. Tsutaya, T. & Yoneda, M. Quantitative Reconstruction of Weaning Ages in Archaeological Human Populations Using Bone Collagen Nitrogen Isotope Ratios and Approximate Bayesian Computation. *PLOS ONE* **8,** e72327 (2013).
55. Wright, L. E. & Schwarcz, H. P. Stable carbon and oxygen isotopes in human tooth enamel: Identifying breastfeeding and weaning in prehistory. *Am. J. Phys. Anthropol.* **106,** 1–18 (1998).
56. Wright, L. E. & Schwarcz, H. P. Correspondence Between Stable Carbon, Oxygen and Nitrogen Isotopes in Human Tooth Enamel and Dentine: Infant Diets at Kaminaljuyú. *J. Archaeol. Sci.* **26,** 1159–1170 (1999).
57. Doumani Dupuy, P. N., Spengler III, R. N. & Frachetti, M. D. Eurasian textiles: Case studies in exchange during the incipient and later Silk Road periods. *Quat. Int.* (2017). doi:10.1016/j.quaint.2016.09.067
58. Mirlas, V., Antonenko, V., Kulagin, V. & Kuldeeva, E. Assessing artificial groundwater recharge on irrigated land using the MODFLOW model: A Case Study from Karatal Agricultural Area, Kazakhstan. *Earth Sci. Res.* **4,** (2015).
59. Mashkour, M. The Subsistence Economy in the Rural Community of Geoktchik Depe in Southern Turkmenistan: Preliminary Results of the Faunal Analysis. in *Archaeozoology of the Near East III, Proceedings of the Third International Symposium on the Archaeozoology of Southwestern Asia and Adjacent Areas* (eds. Buitenhuis, H., Bartosiewicz, L. & Choyke, A. M.) 200–220 (ARC, 1998).
60. Bocherens, H., Mashkour, M., Drucker, D. G., Moussa, I. & Billiou, D. Stable isotope evidence for palaeodiets in southern Turkmenistan during Historical period and Iron Age. *J. Archaeol. Sci.* **33,** 253–264 (2006).
61. Atagarryev, E. *Srednevekobyj Dekhistan*. (Nauka, 1986).
62. Fick, S. E. & Hijmans, R. J. WorldClim 2: new 1-km spatial resolution climate surfaces for global land areas. *Int. J. Climatol.* **37,** 4302–4315 (2017).
63. Chen, F.-H. *et al.* Moisture changes over the last millennium in arid central Asia: a review, synthesis and comparison with monsoon region. *Quat. Sci. Rev.* **29,** 1055–1068 (2010).
64. Hong, B. *et al.* Increasing summer rainfall in arid eastern-Central Asia over the past 8500 years. *Sci. Rep.* **4,** 5279 (2014).
65. Lan, J. *et al.* Climate changes reconstructed from a glacial lake in High Central Asia over the past two millennia. *Quat. Int.* (2017). doi:10.1016/j.quaint.2017.10.035
66. Helama, S., Jones, P. D. & Briffa, K. R. Dark Ages Cold Period: A literature review and directions for future research. *The Holocene* **27,** 1600–1606 (2017).
67. Hengl, T. *et al.* SoilGrids250m: Global gridded soil information based on machine learning. *PLOS ONE* **12,** e0169748 (2017).
68. Oksanen, J. *et al.* vegan: Community Ecology Package. (2017). Available at: https://CRAN.R-project.org/package=vegan.
69. Benjamin, D. J. *et al.* Redefine statistical significance. *Nat. Hum. Behav.* 1 (2017). doi:10.1038/s41562-017-0189-z
70. Bland, J. M. & Altman, D. G. Multiple significance tests: the Bonferroni method. *BMJ* **310,** 170 (1995).
71. Nesbitt, M. Grains. in *The Cultural History of Plants* (eds. Prance, G. & Nesbitt, M.) 45–60 (Routledge, 2005).
72. Handley, L. L. *et al.* The 15N natural abundance (δ15N) of ecosystem samples reflects measures of water availability. *Funct. Plant Biol.* **26,** 185–199 (1999).
73. Bogaard, A. *et al.* Combining functional weed ecology and crop stable isotope ratios to identify cultivation intensity: a comparison of cereal production regimes in Haute Provence, France and Asturias, Spain. *Veg. Hist. Archaeobotany* 1–17 (2015). doi:10.1007/s00334-015-0524-0
74. Fraser, R. A. *et al.* Manuring and stable nitrogen isotope ratios in cereals and pulses: towards a new archaeobotanical approach to the inference of land use and dietary practices. *J. Archaeol. Sci.* **38,** 2790–2804 (2011).
75. Makarewicz, C. A. Winter pasturing practices and variable fodder provisioning detected in nitrogen (δ15N) and carbon (δ13C) isotopes in sheep dentinal collagen. *J. Archaeol. Sci.* **41,** 502–510 (2014).

76. Makarewicz, C. A. Winter is coming: seasonality of ancient pastoral nomadic practices revealed in the carbon (δ13C) and nitrogen (δ15N) isotopic record of Xiongnu caprines. *Archaeol. Anthropol. Sci.* **9,** 405–418 (2017).
77. Szpak, P. Complexities of nitrogen isotope biogeochemistry in plant-soil systems: implications for the study of ancient agricultural and animal management practices. *Front. Plant Sci.* **5,** (2014).
